# Genome-wide analysis of bromodomain gene family in Arabidopsis and rice

**DOI:** 10.3389/fpls.2023.1120012

**Published:** 2023-03-08

**Authors:** T. V. Abiraami, Ravi Prakash Sanyal, Hari Sharan Misra, Ajay Saini

**Affiliations:** ^1^ Molecular Biology Division, Bhabha Atomic Research Centre, Mumbai, Maharashtra, India; ^2^ Homi Bhabha National Institute, Mumbai, Maharashtra, India

**Keywords:** alternative splicing, *Arabidopsis thaliana*, tandem and block duplication, bromodomain, bromodomain-containing genes, homology modeling, *Oryza sativa*, salt-stress response

## Abstract

The bromodomain-containing proteins (BRD-proteins) belongs to family of ‘epigenetic mark readers’, integral to epigenetic regulation. The BRD-members contain a conserved ‘bromodomain’ (BRD/BRD-fold: interacts with acetylated-lysine in histones), and several additional domains, making them structurally/functionally diverse. Like animals, plants also contain multiple Brd-homologs, however the extent of their diversity and impact of molecular events (genomic duplications, alternative splicing, AS) therein, is relatively less explored. The present genome-wide analysis of *Brd*-gene families of *Arabidopsis thaliana* and *Oryza sativa* showed extensive diversity in structure of genes/proteins, regulatory elements, expression pattern, domains/motifs, and the bromodomain (w.r.t. length, sequence, location) among the Brd-members. Orthology analysis identified thirteen ortholog groups (OGs), three paralog groups (PGs) and four singleton members (STs). While more than 40% *Brd*-genes were affected by genomic duplication events in both plants, AS-events affected 60% *A. thaliana* and 41% *O*. *sativa* genes. These molecular events affected various regions (promoters, untranslated regions, exons) of different Brd-members with potential impact on expression and/or structure-function characteristics. RNA-Seq data analysis indicated differences in tissue-specificity and stress response of Brd-members. Analysis by RT-qPCR revealed differential abundance and salt stress response of duplicate *A. thaliana* and *O*. *sativa Brd*-genes. Further analysis of *AtBrd* gene, *AtBrdPG1b* showed salinity-induced modulation of splicing pattern. Bromodomain (BRD)-region based phylogenetic analysis placed the *A. thaliana* and *O*. *sativa* homologs into clusters/sub-clusters, mostly consistent with ortholog/paralog groups. The bromodomain-region displayed several conserved signatures in key BRD-fold elements (α-helices, loops), along with variations (1-20 sites) and indels among the BRD-duplicates. Homology modeling and superposition identified structural variations in BRD-folds of divergent and duplicate BRD-members, which might affect their interaction with the chromatin histones, and associated functions. The study also showed contribution of various duplication events in *Brd*-gene family expansion among diverse plants, including several monocot and dicot plant species.

## 1 Introduction

Gene expression in higher animals and plants is highly complex and regulated at multiple levels in response to cellular/physiological requirements, and unfavorable environmental conditions (Floris et al., 2009; Haak et al., 2017; Merchante et al., 2017; Withers and Dong, 2017). Abiotic stresses negatively affect plant physiology and growth, leading to substantial loss in productivity. Unfavorable environmental conditions (viz. salinity, drought, heat) induces complex transcriptional programing in plants, leading to stress adaptive responses to mitigate detrimental impact (Kreps et al., 2002; Gao et al., 2008). The transcription status of genes is influenced by both genetic and epigenetic components (Singh, 1998; Kim et al., 2015), and unlike the genetic-elements (core promoter, *cis*-elements, enhancers, silencers), the epigenetic controls involve non-sequence-based modifications to alter the expression of genes (Gibney and Nolan, 2010; Lämke and Bäurle, 2017; Rendina González et al., 2018). Epigenetic modifications of DNA and/or histones affect the state of chromatin and transcription activity (Iwasaki and Paszkowski, 2014), leading to the enhanced response potential of the genetic material (Strahl and Allis, 2000; Loidl, 2004). Post-translational modifications (PTMs: acetylation, methylation, phosphorylation, ubiquitylation etc.) affect the characteristics of several cellular proteins including histones, where such modifications modulate the nucleosome dynamics (Bowman and Poirier, 2015). Among these, acetylation of lysine residues plays important roles in protein-protein interactions, nuclear transport, as well as modulation of chromatin state due to impact on positive charge and steric bulk of a nucleosome (Bowman and Poirier, 2015). The cellular epigenetic regulation is based on a system of ‘writer’, ‘reader’ and ‘eraser’ proteins for dynamic management of PTM marks (Musselman et al., 2012; Zhao et al., 2018). For acetylation/deacetylation of lysine residues, lysine acetyltransferases (KATs) and lysine deacetylases/histone deacetylases (KDAC/HDACs) perform ‘writer’ and ‘eraser’ functions (Pandey et al., 2002; Musselman et al., 2012), while the conserved bromodomain (BRD/BRD-fold), an important component of several chromatin-associated proteins, serve as ‘reader’ of lysine acetylation on histones (Drazic et al., 2016).

The bromodomain containing genes (*Brd*-genes) were first identified in the *Drosophila melanogaster* (Tamkun et al., 1992), and subsequently reported in diverse eukaryotes (Rao et al., 2014). The ~110 amino acid bromodomain (BRD)-region folds into four α-helices (αZ, αA, αB, αC) connected by three loops (ZA, AB, BC) to form a conserved BRD/BRD-fold (a hydrophobic pocket) to recognize acetylated lysine residues in the histones (Marmorstein and Berger, 2001; Bottomley, 2004; Mujtaba et al., 2007; Ferri et al., 2016). A single BRD-domain is capable of recognizing acetylated lysine residues on different histones (Josling et al., 2012). BRD-containing proteins (alone or as multi-protein complexes) are involved in regulation of gene expression by different mechanisms viz. chromatin remodeling, histone modifications, transcriptional machinery regulation (Florence and Faller, 2001; Fujisawa and Filippakopoulos, 2017). The *Brd*-gene family, which is a large and diverse family among different organisms, contains a total of 46 Brd-members in humans, divided into eight structurally and functionally distinct groups (Filippakopoulos et al., 2012). Human Brd-members have been studied well for their involvement in chromatin dynamics and diverse cellular functions, and have gained attention as promising drug targets for different disease conditions (Zeng and Zhou, 2002; Sanchez and Zhou, 2009; Taniguchi, 2016; Cochran et al., 2019; Uppal et al., 2019; Boyson et al., 2021).

Epigenetic-changes in chromatin structure and organization, and its impact on expression of genes is equally important for cellular and physiological requirements, and stress responses in plants. Salinity is a complex condition, which along with ionic imbalance-mediated toxicity, also leads to osmotic and oxidative stress, and hence multiple mechanisms including epigenetic regulation are activated (Fransz and De Jong, 2002; Rosa and Shaw, 2013; Kim et al., 2015; Vergara and Gutierrez, 2017; Bhadouriya et al., 2021; Pei et al., 2021; Yung et al., 2021). Like animals, plants also harbor large *Brd*-gene families, however the extent of divergence of Brd-members, and their functional significance (and role of genomic duplications and alternative splicing events) is relatively less explored. Studies on few Brd-homologs from Arabidopsis and other plants have shown their involvement in functions like seed germination (Duque and Chua, 2003), leaf development (Chua et al., 2005), mitotic cell cycle (Airoldi et al., 2010), sugar and abscisic acid responses (Misra et al., 2018), growth and development (Rao et al., 2014; Martel et al., 2017), pathogen perception and immune response (Sukarta et al., 2020; Zhou et al., 2022), and as important subunits of SWI/SNF chromatin remodelers (Jarończyk et al., 2021).

The number of *Brd*-gene family members varies in different organisms (Rao et al., 2014). An important feature of most plants genomes is genomic duplication events that have contributed towards generation of additional copies of several genes, leading to divergence towards regulatory, structural and functional differences (Flagel and Wendel, 2009; Barker et al., 2012; Qiao et al., 2019). In addition to the diversity of genes in multi-member families, it is also important to identify the conserved orthologs as well as species-specific paralogs to get insights into the evolutionary trends, and lineage-specific events (Altenhoff et al., 2019). Further, a large number of reports have shown the involvement of alternative splicing (AS) mechanism in regulation of expression of genes in diverse conditions, and alteration of key features of the alternative protein isoforms (Reddy et al., 2013). Involvement of both these mechanisms on diversity of *Brd*-genes among plants has not been explored well, and is worth investigating.

In the present study, we carried out genome-wide analysis of *Brd*-gene family in model plants *A. thaliana* (dicot) and *O. sativa* (monocot), to understand the extent of diversity of genes and proteins, regulatory regions, tissue- and stress-induced expression and splicing dynamics, domains-motifs architecture, and variations in the BRD-fold. Analysis of conserved orthologs, species-specific paralogs and singleton Brd-members, revealed the differential evolutionary trend of *Brd*-genes in the two species. Result also showed the effect of genome duplication and AS-events on the characteristics of *Brd* genes, proteins as well as the BRD-fold, in both the species. Moreover, analysis also showed substantial contribution of various duplication events in *Brd*-gene copy number increase among lower and higher plants, including monocot and dicot species. To our knowledge, this is the first study on analysis of diversity of Brd-homologs of model plants, *A. thaliana* and *O. sativa*, which will be useful for further studies on deciphering their functional significance in chromatin dynamics and response to diverse cellular conditions and stress responses.

## 2 Materials and methods

### 2.1 Identification of bromodomain genes in *A. thaliana* and *O. sativa* genome databases

Multiple databases were used for retrieval of sequence data of *Brd*-genes from *A. thaliana* (referred to as ‘*AtBrd*’) and *O. sativa* (referred to as ‘*OsBrd*’). The Arabidopsis Information Resource (TAIR, https://www.arabidopsis.org/index.jsp) and PLAZA dicots (https://bioinformatics.psb.ugent.be/plaza/versions/plaza _v4_5_dicots/, Van Bel et al., 2018) databases were used for *A. thaliana*, and for *O. sativa*, Rice Genome Annotation Project (RGAP, http://rice.uga.edu/) and PLAZA monocots (version 4.5, https://bioinformatics.psb.ugent.be/plaza/versions/plaza_v4_5_monocots/, Van Bel et al., 2018) databases were used. Analysis based on Brd-family IDs (SCOP database ID: Brd- superfamily, 3001843; bromodomain family, 4000871) was used for identification of Brd-family members, and confirmation was also done for presence of bromodomain at Conserved Domain Database (CDD, NCBI, https://www.ncbi.nlm.nih.gov/cdd/).

### 2.2 *In silico* analysis of characteristics of genes, proteins and transcripts

The structure and organization of *AtBrd* and *OsBrd* genes in terms of untranslated regions (UTRs), exons, and introns was analyzed using the Gene Structure Display Server (GSDS, http://gsds.gao-lab.org/, Hu et al., 2015). The important characteristics (molecular weight, MW; isoelectric point, pI etc.) of the AtBRD and OsBRD proteins were estimated using the ProtParam tool on the ExPASy website (http://web.expasy.org/protparam/). The alternative isoforms of the *AtBrd* and *OsBrd* genes were retrieved from the respective databases, and compared with the corresponding constitutive isoforms by pair-wise alignment using ClustalX (Thompson et al., 1997).

### 2.3 Identification of orthologs and paralogs

For the identification of orthologs and paralogs among the *A. thaliana* and *O. sativa* Brd-family members, OrthoVenn2 online tool was used (https://orthovenn2.bioinfotoolkits.net/home, Xu et al., 2019). In brief, the full-length sequences of BRD-containing sequences were analysed at OrthoVenn2 portal (E-value, 1e-2; inflation value, 1.5) to identify the shared ortholog groups (OGs, representation of both species), species-specific paralog groups (PGs, representation of one species) and singleton sequences (STs, not part of ortholog/paralog groups).

### 2.4 *In silico* analysis of promoter structure and *cis-*regulatory elements

Upstream regulatory regions (up to 2000 bp) of *AtBrd* and *OsBrd* genes were retrieved from the TAIR, RGAP and PLAZA databases. Presence and organization of CpG islands, transcription factor binding sites (TFBS), and tandem repeats motifs was analyzed at Plant Promoter Analysis Navigator online resource (PlantPAN3.0, http://plantpan3.itps.ncku.edu.tw/), whereas *cis-*elements (types, location, copy number) were analyzed at Plant *Cis*-Acting Regulatory Elements databases (PlantCARE, http://bioinformatics.psb.ugent.be/webtools/plantcare/html/).

### 2.5 *In silico* analysis of conserved domains, functional sites and motifs

Presence of conserved domains, important functional sites in the BRD proteins was analysed at CDD-NCBI and PROSITE (https://prosite.expasy.org/, Sigrist et al., 2012). Domain analysis was carried out using default search parameters and only significant hits were considered for analysis. Conserved motifs were analysed at MEME online Suite (version 5.4.1, http://meme-suite.org/tools/meme, Bailey et al., 2015) using following parameters; minimum and maximum motif width: 6-50, number of motifs: 15.

### 2.6 *In silico* analysis of gene expression using RNA-Seq data

The RNA-Seq expression data (as FPKM values, Fragments Per Kilobase of transcript, per Million mapped reads) of respective *Brd*-genes was retrieved from the Arabidopsis RNA-seq Database (V2, http://ipf.sustech.edu.cn/pub/athrna/) for different tissues (shoot, root, stem, meristem, seedling, embryo, leaf, silique, endosperm, seed, flower and pollen) and two stress conditions (cold and drought). Rice Expression Database (http://expression.ic4r.org/) was used for retrieving data for rice tissues (root, shoot, panicle (3 stages), anther (2 stages), pistil, aleurone and seed) and two stress conditions (drought and cadmium). The gene/locus names were used for search and retrieval of FPKM data. In case of multiple libraries, the average FPKM values were used for analysis. FPKM values were log2-transformed and used for generation of heat map-based transcript profiles by Heatmap Illustrator software (HemI, version 1.0, Deng et al., 2014).

### 2.7 Plant growth conditions, total RNA isolation, cDNA synthesis and RT-qPCR analysis

Seeds of *A. thaliana* ecotype Columbia-0 (Col-0) were grown on MS-agar plates containing germination media (HiMedia, India), in Sanyo MLR-351H plant growth chamber (temperature: 23 ± 1°C, photoperiod settings: 14 h light/10 h dark). For salt-stress treatment, 15-day old seedlings were transferred to MS-media containing 150 mM sodium chloride (NaCl). Seeds of rice genotype NSICRc106 (obtained from International Rice Research Institute, Philippines) were grown in Hoagland media (Himedia, India) in Sanyo MLR-351H plant growth chamber as detailed previously (Sanyal et al., 2018). Six-day-old seedlings were subjected to salt-stress (150 mM NaCl). Tissue samples of both the plants were collected at 24 h time-point, frozen in liquid nitrogen, and stored at -70 °C. Three-five seedlings were pooled for total RNA isolation by TRIzol (Invitrogen, USA), which was assessed for quality and quantity, and treated with DNase I (Roche Diagnostics, Germany) to remove DNA contamination. Total RNA (10 µg) was reverse transcribed using SuperScript II reverse transcriptase (Invitrogen, USA) using anchored oligo(dT)23 and random nonamers (New England Biolabs, USA), as per the protocol recommended by the manufacturer.

Transcript levels of *Brd*-genes (six *AtBrd*-gene pairs and five *OsBrd*-gene pairs), and constitutive and alternative splice variants of one *AtBrd*-gene (*AtBrdPG1b*) were analyzed by RT-qPCR analysis, using oligonucleotide primers designed utilizing the exon-intron information available from RGAP and TAIR databases (**Supplementary Table 1**). Briefly, RT-qPCR assays were carried out on LightCycler LC480 II (Roche Diagnostics, Germany) using SYBR Green Jumpstart *Taq* Ready mix (Sigma-Aldrich, USA) using following cycling settings: 94 °C (2 min), 45 cycles of 94 °C (15 sec), 60 °C (15 sec), 68 °C (20 sec), followed by melting curve analysis to assess the amplicon specificity. Three independent replicate sets were used, and analysis was carried out as per Schmittgen and Livak (2008) using *AtActin* and *OselF1α* as reference genes. Statistical analysis was carried out by Student’s t-test and differences were considered significant only when the P < 0.05.

### 2.8 Multiple sequence alignment and phylogenetic analysis

Multiple sequence alignment of the bromodomain (BRD) region of AtBRDs and OsBRDs was done by ClustalX, and used for estimation of sequence divergence, and analysis of genetic relationships using neighbour-joining approach (Saitou and Nei, 1987) in Molecular Evolutionary Genetic Analysis X software (MEGAX, version 10.0.5, Kumar et al., 2018). Statistical analysis was carried out by bootstrap method (Felsenstein, 1985). To identify the conserved residues in key elements of BRD-fold, the alignment was transformed into a sequence logo using TBtools (Chen et al., 2020). In a separate analysis, BRD regions of few human homologs containing single (UniProt accession numbers: Q9NR48, ASH1L; Q9NPI1, BRD7; Q9H0E9-2, BRD8B; P55201-1, BRPF1A; Q92830, GCN5L2; Q03164, MLL; Q13342, SP140; Q13263, TRIM28; Q9UPN9, TRIM33A; O15016, TRIM66; P51531, SMCA2; P51532, SMCA4) or two bromodomains (P25440, BRD2; Q15059, BRD3; O60885, BRD4; Q58F21, BRDT; Q6RI45, BRWD3; P21675, TAF1; Q9NS16, WDR9) were also included.

### 2.9 Homology modelling and comparison

The homology models of BRD-fold of several *A. thaliana* and *O. sativa* BRD proteins were generated at SWISS-MODEL workspace (http://swissmodel.expasy.org) using automated mode option, and compared for structural differences. For identification of structural differences due to variations among BRD-folds, the homology models of BRD-folds of duplicate Brd-pairs or divergent Brd-members were superposed using structure comparison tools at SWISS-MODEL workspace.

### 2.10 Analysis of duplication events among plant genomes

The *Brd*-gene members from *A. thaliana* and *O. sativa* were analyzed for block and tandem duplication events at PLAZA (version 4.5) dicots and monocots databases (https://bioinformatics.psb.ugent.be/plaza/, Van Bel et al., 2018). InterPro identifier IPR001487 (bromodomain) was used to identify the chromosomal locations of all *Brd*-genes (including duplicate-pairs), and represented using the Circle Plot tool available at PLAZA server. Additional 79 plant genomes including seven lower photosynthetic organisms, 27 monocots and 45 dicots (available at PLAZA monocots and dicots databases) were analyzed for prevalence of different duplication events (block, tandem, combined tandem + block events) leading to multiple *Brd*-genes.

## 3 Results

### 3.1 Diversity of Brd-members in *A. thaliana* and *O. sativa*: Block and tandem duplications

**Figure 1:**
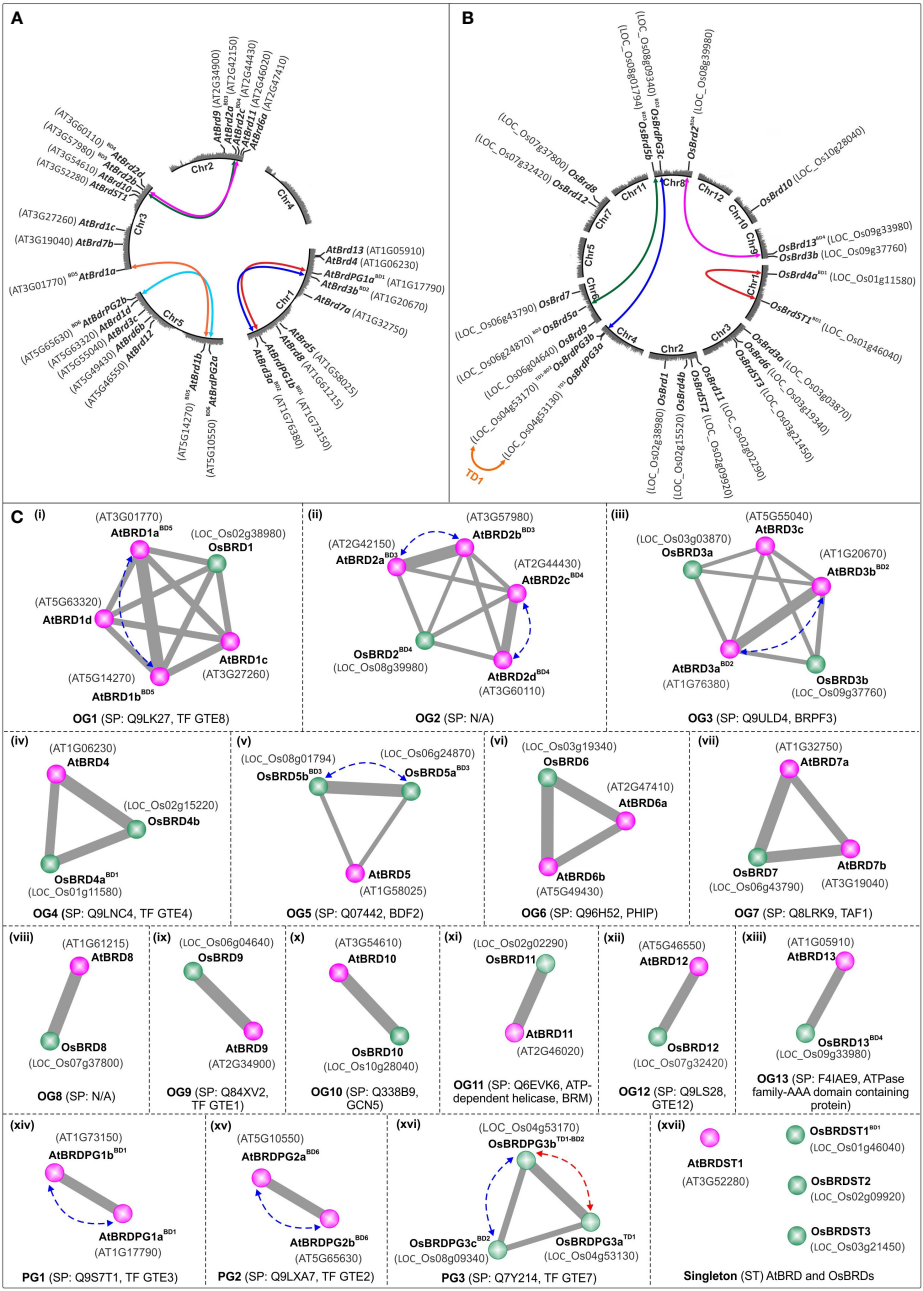
Circle-plot representation of chromosomal distribution of *Brd*-genes in *A. thaliana* **(A)** and *O. sativa* **(B)** genomes. The gene designations are indicated in bold font, while the locus numbers, as per TAIR (for *AtBrds*) and RGAP (for *OsBrds*) databases, are given in the parenthesis. Colored connecting lines indicate the tandem/block-duplicated *Brd*-genes, ‘Chr1-5 (*A. thaliana*)/Chr1-12 (*O. sativa*)’ indicate chromosome numbers, ‘BD’ and ‘TD’ indicate block and tandem duplication events. **(C)** Conserved ortholog groups (OGs), paralog groups (PGs), and singleton BRD-members (STs), in *A. thaliana* (purple circles) and *O. sativa* (green circles), as per orthology analysis at Orthovenn2 server (https://orthovenn2.bioinfotoolkits.net/). Blue and red colored dotted lines indicate the block-duplicated (BD, blue line) and tandem-duplicated (TD, red line) Brds, while the functional information (based on SwissProt IDs) is indicated in the parenthesis (N/A indicates ‘No Hit’).

Database analysis identified a total of 28 *Brd*-gene family members in *A. thaliana* and 22 members in *O. sativa* (**Tables 1**, **2**). The *Brd*-genes displayed unequal chromosomal distribution in both *A. thaliana* (Chr1: 09, Chr3/Chr5: 07 each, Chr2: 05, Chr4: nil; **Table 1** and **Figure 1A**) and *O. sativa* (Chr2: 04, Chr3/Chr6/Chr8: 03 each, Chr1/Chr4/Chr7/Chr9: 02 each, Chr10: 01, Chr5/Chr11/Chr12: nil; **Table 2** and **Figure 1B**). The *Brd*-genes and encoded proteins in both the species showed extensive diversity in characteristics viz. number of alternative isoforms, molecular weight, isoelectric point (**Tables 1**, **2**).

**Table 1:** An overview of characteristics of 28 bromodomain-containing genes (*Brd*-genes) in *Arabidopsis thaliana* genome.

Sr. No.	Locus No^1^, Designation^2^ and OG/PG/ST category^3^	Chr No	Gene Length (bp)	Number of Transcripts, IDs and (Designation)		CDS Length (bp)	Protein Length (aa)	Molecular Weight (Da)	Isoelectric point (pI)
1	AT1G05910 (*AtBrd13*), OG13	1	6500	1	AT1G05910.1 (*AtBrd13.1*)	3633	1210	133782.5	5.64
2	AT1G06230 (*AtBrd4*), OG4		4094	4	AT1G06230.1 (*AtBrd4.1*)	2301	766	84093.9	5.01
					AT1G06230.2 (*AtBrd4.2*)*	2301	766	84093.9	5.01
					AT1G06230.3 (*AtBrd4.3*)*	2301	766	84093.9	5.01
					AT1G06230.4 (*AtBrd4.4*)*	2301	766	84093.9	5.01
3	AT1G17790 (*AtBrdPG1a*)^BD1^, PG1		2290	1	AT1G17790.1 (*AtBrdPG1a.1*)	1464	487	53454.3	6.66
4	AT1G20670 (*AtBrd3b*)^BD2^, OG3		3960	1	AT1G20670.1 (*AtBrd3b.1*)	1959	652	72955.5	6.86
5	AT1G32750 (*AtBrd7a*), OG7		10519	1	AT1G32750.1 (*AtBrd7a.1*)	5760	1919	217191.7	5.55
6	AT1G58025 (*AtBrd5*), OG5		4577	6	AT1G58025.1 (*AtBrd5.1*)	1719	572	64849.3	6.80
					AT1G58025.2 (*AtBrd5.2*)***	1749	582	66001.7	6.63
					AT1G58025.3 (*AtBrd5.3*)***	1722	573	64920.4	6.80
					AT1G58025.4 (*AtBrd5.4*)***	1722	573	64920.4	6.80
					AT1G58025.5 (*AtBrd5.5*)***	1722	573	64920.4	6.80
					AT1G58025.6 (*AtBrd5.6*)*	1719	572	64849.3	6.80
7	AT1G61215 (*AtBrd8*), OG8		2975	2	AT1G61215.1 (*AtBrd8.1*)	1428	475	52706.5	10.29
					AT1G61215.2 (*AtBrd8.2*)***	1371	456	50768.3	10.44
8	AT1G73150 (*AtBrdPG1b*)^BD1^, PG1		2434	2	AT1G73150.1 (*AtBrdPG1b.1*)	1386	461	50811.8	6.29
					AT1G73150.2 (*AtBrdPG1b.2*)***	1299	432	48308.8	6.66
9	AT1G76380 (*AtBrd3a*)^BD2^, OG3		3592	3	AT1G76380.1 (*AtBrd3a.1*)	1740	579	64850.0	7.43
					AT1G76380.2 (*AtBrd3a.2*)***	1743	580	64907.1	7.43
					AT1G76380.3 (*AtBrd3a.3*)**	1740	579	64792.0	7.80
10	AT2G34900 (*AtBrd9)*, OG9	2	2672	2	AT2G34900.1 (*AtBrd9.1*)	1161	386	43441.9	6.30
					AT2G34900.2 (*AtBrd9.2*)***	831	276	31625.9	8.37
11	AT2G42150 (*AtBrd2a*)^BD3^, OG2		2375	1	AT2G42150.1 (*AtBrd2a.1*)	1896	631	70445.1	8.61
12	AT2G44430 (*AtBrd2c*)^BD4^, OG2		2995	1	AT2G44430.1 (*AtBrd2c.1*)	1941	646	72404.9	8.86
13	AT2G46020 (*AtBrd11*), OG11		9523	6	AT2G46020.1 (*AtBrd11.1*)	6579	2192	245437.4	9.30
					AT2G46020.2 (*AtBrd11.2*)***	6582	2193	245467.4	9.23
					AT2G46020.3 (*AtBrd11.3*)***	6582	2193	245467.4	9.23
					AT2G46020.4 (*AtBrd11.4*)***	6582	2193	245467.4	9.23
					AT2G46020.5 (*AtBrd11.5*)*	6579	2192	245437.4	9.30
					AT2G46020.6 (*AtBrd11.6*)*	6579	2192	245437.4	9.30
14	AT2G47410 (*AtBrd6a*), OG6		9156	6	AT2G47410.1 (*AtBrd6a.1*)	4563	1520	171534.9	7.07
					AT2G47410.2 (*AtBrd6a.2*)***	4560	1519	171447.9	7.07
					AT2G47410.3 (*AtBrd6a.3*)***	4047	1348	151704.5	7.05
					AT2G47410.4 (*AtBrd6a.4*)***	4017	1338	150692.5	7.11
					AT2G47410.5 (*AtBrd6a.5*)**	4593	1530	172546.9	7.02
					AT2G47410.6 (*AtBrd6a.6*)***	4047	1348	151704.5	7.05
15	AT3G01770 (*AtBrd1a*)^BD5^, OG1	3	3509	2	AT3G01770.1 (*AtBrd1a.1*)	1863	620	69880.7	5.11
					AT3G01770.2 (*AtBrd1a.2*)***	1470	489	54637.2	6.74
16	AT3G19040 (*AtBrd7b*), OG7		8593	2	AT3G19040.1 (*AtBrd7b.1*)	5361	1786	202250.4	7.66
					AT3G19040.2 (*AtBrd7b.2*)***	5379	1792	202813.7	7.37
17	AT3G27260 (*AtBrd1c*), OG1		5232	4	AT3G27260.1 (*AtBrd1c.1*)	2442	813	90232.5	4.53
					AT3G27260.2 (*AtBrd1c.2*)***	2295	764	85090.1	4.58
					AT3G27260.3 (*AtBrd1c.3*)***	2469	822	91181.7	4.52
					AT3G27260.4 (*AtBrd1c.4*)**	2157	718	79762.2	4.75
18	AT3G52280 (*AtBrdST1*), ST1		2455	2	AT3G52280.1 (*AtBrdST1.1*)	1110	369	42392.4	8.01
					AT3G52280.2 (*AtBrdST1.2*)***	1161	386	44382.7	8.56
19	AT3G54610 (*AtBrd10*), OG10		4248	1	AT3G54610.1 (*AtBrd10.1*)	1707	568	63123.0	6.42
20	AT3G57980 (*AtBrd2b*)^BD3^, OG2		2504	2	AT3G57980.1 (*AtBrd2b.1*)	1953	650	72310.1	9.86
					AT3G57980.2 (*AtBrd2b.2*)***	1959	652	72551.4	9.86
21	AT3G60110 (*AtBrd2d*)^BD4^, OG2		3923	1	AT3G60110.1 (*AtBrd2d.1*)	1926	641	72045.1	9.74
22	AT5G10550 (*AtBrdPG2a*)^BD6^, PG2	5	1919	1	AT5G10550.1 (*AtBrdPG2a.1*)	1746	581	64102.7	6.55
23	AT5G14270 (*AtBrd1b*)^BD5^, OG1		4314	3	AT5G14270.1 (*AtBrd1b.1*)	2067	688	75894.3	4.61
					AT5G14270.2 (*AtBrd1b.2*)***	2070	689	75991.4	4.61
					AT5G14270.3 (*AtBrd1b.3*)*	2067	688	75894.3	4.61
24	AT5G46550 (*AtBrd12*), OG12		2743	1	AT5G46550.1 (*AtBrd12.1*)	1485	494	55618.7	9.82
25	AT5G49430 (*AtBrd6b*), OG6		9715	2	AT5G49430.1 (*AtBrd6b.1*)	5034	1677	186918.3	7.08
					AT5G49430.2 (*AtBrd6b.2*)*	5034	1677	186918.3	7.08
26	AT5G55040 (*AtBrd3c*), OG3		5139	2	AT5G55040.1 (*AtBrd3c.1*)	2751	916	103414.1	6.00
					AT5G55040.2 (*AtBrd3c.2*)*	2751	916	103414.1	6.00
27	AT5G63320 (*AtBrd1d*), OG1		5343	3	AT5G63320.1 (*AtBrd1d.1*)	3186	1061	118972.0	4.43
					AT5G63320.2 (*AtBrd1d.2*)***	1434	477	53000.4	7.20
					AT5G63320.3 (*AtBrd1d.3*)***	1434	477	53000.4	7.20
28	AT5G65630 (*AtBrdPG2b*)^BD6^, PG2		3743	1	AT5G65630.1 (*AtBrdPG2b.1*)	1773	590	65076.7	6.70

^1^The locus numbers are as per TAIR database (The Arabidopsis Information Resource at https://www.arabidopsis.org/); ^2^Simplified designation of the genes as per clustering in different ortholog/paralog groups or singleton category; ^3^Ortholog group (OG)/paralog group (PG)/singleton (ST) category association of Brd-members; BD1-6: Block duplication events 1-6; Chr No: Chromosome number; CDS: Coding DNA sequence; Alternatively spliced transcripts with differences in UTR (*) exon (**) or both regions (***) are indicated.

**Table 2:** An overview of characteristics of 22 bromodomain-containing genes (*Brd*-genes) in *Oryza sativa* genome.

Sr. No.	Locus No^1^, Designation^2^ and OG/PG/ST category^3^	Chr No	Gene Length (bp)	Number of Transcripts, Transcript IDs and (Designation)		CDS Length (bp)	Protein Length (aa)	Molecular Weight (Da)	Isoelectric Point (pI)
1	LOC_Os01g11580 (*OsBrd4a*) ^BD1^, OG4	1	5988	2	LOC_Os01g11580.1 (*OsBrd4a.1*)	1068	355	39749.8	4.96
					LOC_Os01g11580.2 (*OsBrd4a.2*)***	663	220	24655.7	4.42
2	LOC_Os01g46040 (*OsBrdST1*) ^BD1^, ST		4046	1	LOC_Os01g46040.1 (*OsBrdST1.1*)	717	238	26206.1	6.95
3	LOC_Os02g02290 (*OsBrd11*), OG11	2	10570	1	LOC_Os02g02290.1 (*OsBrd11.1*)	6603	2200	246212.0	9.07
4	LOC_Os02g09920 (*OsBrdST2*), ST		5584	1	LOC_Os02g09920.1 (*OsBrdST2.1*)	2940	979	110342.0	4.79
5	LOC_Os02g15220 (*OsBrd4b*), OG4		7999	3	LOC_Os02g15220.1 (*OsBrd4b.1*)	1971	656	71476.3	10.00
					LOC_Os02g15220.2 (*OsBrd4b.2*)*	1971	656	71476.3	10.00
					LOC_Os02g15220.4 (*OsBrd4b.4*)***	1875	624	68946.6	10.20
6	LOC_Os02g38980 (*OsBrd1*), OG1		5209	5	LOC_Os02g38980.1 (*OsBrd1.1*)	2145	714	78630.4	4.84
					LOC_Os02g38980.3 (*OsBrd1.3*)***	1728	575	63133.5	5.62
					LOC_Os02g38980.4 (*OsBrd1.4*)***	1701	566	62093.3	5.61
					LOC_Os02g38980.5 (*OsBrd1.5*)***	1443	480	52786.3	6.67
					LOC_Os02g38980.6 (*OsBrd1.6*)***	1302	433	48256.5	8.01
7	LOC_Os03g03870 (*OsBrd3a*), OG3	3	6059	1	LOC_Os03g03870.1 (*OsBrd3a.1*)	1452	483	52387.9	8.43
8	LOC_Os03g19340 (*OsBrd6*), OG6		16548	1	LOC_Os03g19340.1 (*OsBrd6.1*)	4881	1626	183000	6.64
9	LOC_Os03g21450 (*OsBrdST3*), ST		2605	1	LOC_Os03g21450.1 (*OsBrdST3.1*)	1677	558	59620.1	10.63
10	LOC_Os04g53130 (*OsBrdPG3a*) ^TD1^, PG3	4	3427	1	LOC_Os04g53130.1 (*OsBrdPG3a.1*)	1068	355	39412.4	4.81
11	LOC_Os04g53170 (*OsBrdPG3b*) ^TD1-BD2^, PG3		2150	1	LOC_Os04g53170.1 (*OsBrdPG3b.1*)	1371	456	50290.7	6.94
12	LOC_Os06g04640 (*OsBrd9*), OG9	6	5300	3	LOC_Os06g04640.1 (*OsBrd9.1*)	1083	360	40658.1	7.05
					LOC_Os06g04640.2 (*OsBrd9.2*)***	819	272	31690.2	6.19
					LOC_Os06g04640.3 (*OsBrd9.3*)***	684	227	26418.0	6.32
13	LOC_Os06g24870 (*OsBrd5a*) ^BD3^, OG5		5765	3	LOC_Os06g24870.1 (*OsBrd5a.1*)	1137	378	40896.8	8.15
					LOC_Os06g24870.2 (*OsBrd5a.2*)*	1137	378	40896.8	8.15
					LOC_Os06g24870.3 (*OsBrd5a.3*)*	1137	378	40896.8	8.15
14	LOC_Os06g43790 (*OsBrd7*), OG7		14210	1	LOC_Os06g43790.1 (*OsBrd7.1*)	5475	1824	206046	5.46
15	LOC_Os07g32420 (*OsBrd12*), OG12	7	8036	3	LOC_Os07g32420.1 (*OsBrd12.1*)	1455	484	53992.3	8.33
					LOC_Os07g32420.2 (*OsBrd12.2*)*	1455	484	53992.3	8.33
					LOC_Os07g32420.3 (*OsBrd12.3*)***	921	306	33587.9	9.58
16	LOC_Os07g37800 (*OsBrd8*), OG8		4117	3	LOC_Os07g37800.1 (*OsBrd8.1*)	1485	494	53351.1	10.54
					LOC_Os07g37800.2 (*OsBrd8.2*)**	1407	468	50177.5	9.83
					LOC_Os07g37800.3 (*OsBrd8.3*)***	1035	344	37209.5	8.15
17	LOC_Os08g01794 (*OsBrd5b*) ^BD3^, OG5	8	7581	2	LOC_Os08g01794.1 (*OsBrd5b.1*)	1773	590	65099.3	5.49
					LOC_Os08g01794.2 (*OsBrd5b.2*)*	1773	590	65099.3	5.49
18	LOC_Os08g09340 (*OsBrdPG3c*) ^BD2^, PG3		2119	2	LOC_Os08g09340.1 (*OsBrdPG3c.1*)	1446	481	53638.1	6.63
					LOC_Os08g09340.2 (*OsBrdPG3c.2*)***	1260	419	47311.1	9.65
19	LOC_Os08g39980 (*OsBrd2*) ^BD4^, OG2		3367	1	LOC_Os08g39980.1 (*OsBrd2.1*)	1983	660	68668.1	9.38
20	LOC_Os09g33980 (*OsBrd13*) ^BD4^, OG13	9	7027	1	LOC_Os09g33980.1 (*OsBrd13.1*)	3597	1198	133903	6.49
21	LOC_Os09g37760 (*OsBrd3b*), OG3		5783	1	LOC_Os09g37760.1 (*OsBrd3b.1*)	1245	414	45957.8	9.84
22	LOC_Os10g28040 (*OsBrd10*), OG10	10	6439	1	LOC_Os10g28040.1 (*OsBrd10.1*)	1536	511	56685.3	6.79

^1^The locus numbers are as per RGAP database (Rice Genome Annotation Project at http://rice.uga.edu/); ^2^Simplified designation of the genes as per clustering in different ortholog/paralog groups or singleton category; ^3^Ortholog group (OG)/paralog group (PG)/singleton (ST) category association of Brd-members; BD1-4: Block duplication events 1-4; TD1: Tandem duplication event; Chr No: Chromosome number; CDS: Coding DNA sequence; Alternatively spliced transcripts with differences in UTR; (*) exon (**) or both regions (***) are indicated.

Syntenic analysis identified that in *A. thaliana* six *AtBrd*-gene pairs have originated due to block duplication events (BD1-BD6) (**Figure 1A**), including two intra-chromosomal duplications in Chr1 (BD1: AT1G17790-AT1G73150 and BD2: AT1G20670-AT1G76380) and one in Chr5 (BD6: AT5G10550-AT5G65630). Remaining duplicated *AtBrd*-gene pairs involved inter-chromosomal block duplications viz. BD3 (Chr2-Chr3, AT2G42150-AT3G57980), BD4 (Chr2-Chr3, AT2G44430-AT3G60110) and BD5 (Chr3-Chr5, AT3G01770-AT5G14270) (**Figure 1A**). *Oryza sativa* genome harbored five *OsBrd*-gene pairs, originated due to one tandem (TD1) and four block duplication events (BD1-BD4), of which one was an intra-chromosomal event in Chr1 (BD1: LOC_Os01g11580-LOC_Os01g46040) and three inter-chromosomal events viz. BD2 (Chr4-Chr8, LOC_Os04g53170-LOC_Os08g09340), BD3 (Chr6-Chr8, LOC_Os06g24870-LOC_Os08g01794) and BD4 (Chr8-Chr9, LOC_Os08g39980-LOC_Os09g33980) (**Figure 1B**). In addition, LOC_Os04g53170 (involved in BD2 event) was also involved in a tandem duplication (TD1) event leading to LOC_Os04g53130 on Chr4 (**Figure 1B**).

### 3.2 Orthologs and paralogs among *A. thaliana* and *O. sativa* Brd-members

Analysis of 28 AtBRDs and 22 OsBRDs at OrthoVenn2 server identified 13 ortholog groups (OG1-OG13), three paralog groups (PG1-PG3), and four singleton (ST) members (one AtBRD, three OsBRDs) (**Figure 1C, i - xvii**). For subsequent description, the Brd-members were designated using a simplified scheme based on five-components, indicative of their OG/PG/ST association and duplication status: 1) At/Os (species, At: *A. thaliana* and Os: *O. sativa*), 2) *Brd*/BRD (gene, transcript/protein), 3) OG1-13/PG1-3/ST1-3 (for OG/PG/ST), 4) a-d (multiple members in a group), and 5) BD/TD (block/tandem duplication event). For example, OG1 cluster contains one OsBRD (designated as OsBRD1) and four AtBRDs (designated as AtBRD1a^BD5^, AtBRD1b ^BD5^, AtBRD1c, and AtBRD1d), of which two (AtBRD1a^BD5^and AtBRD1b ^BD5^) are outcome of block duplication event, BD5 (**Figure 1C-i**). This scheme was useful to compare the evolutionary trend of Brd-members in the two species (**Figure 1C**).

The OGs showed variable representation of species-specific Brd-members and displayed different configurations relative to AtBRD vs osBRD-members viz. many-to-one (OG1, OG2, OG6, OG7; **Figure 1C-i, 1C-ii, 1C-vi, 1C-vii**), many-to-many (OG3, **Figure 1C-iii**), one-to-many (OG4, OG5, **Figure 1C-iv, 1C-v**), and one-to-one (OG8-OG13, **Figure 1C-viii** to **1C-xiii**). Paralog groups PG1, PG2 were specific to *A. thaliana* (**Figure 1C-xiv, 1C-xv**), and PG3 was specific to *O. sativa* (**Figure 1C-xvi**). Certain OGs/PGs harboured multiple Brd-members due to species-specific block/tandem duplication events, viz. OG1 (BD5, **Figure 1C-i**), OG2 (BD3, BD4, **Figure 1C-ii**), OG3 (BD2, **Figure 1C-iii**), OG5 (BD3, **Figure 1C-v**), PG1 (BD1, **Figure 1C-xiv**), PG2 (BD6, **Figure 1C-xv**), PG3 (block and tandem events in *O.* sativa, BD2 and TD1, **Figure 1C-xvi**). Interestingly, OsBRD-members of duplication events BD1 (OsBRD4a-OsBRDST1) and BD4 (OsBRD2-OsBRD13) clustered in different groups, suggesting relatively primitive events. The analysis identified conserved functions specific to different clusters viz. OG1 (transcription factor, TF GTE8), OG3 (bromodomain and PHD finger-containing protein 3, BRPF3), OG4 (transcription factor, TF GTE4), OG5 (bromodomain-containing factor, BDF2), OG6 (PH interacting protein, PHIP), OG7 (transcription initiation factor TF11D subunit 1, TAF1), OG9 (transcription factor, TF GTE1), OG10 (histone acetyltransferase, GCN5), OG11 (ATP dependent helicase, BRM a subunit of SWI/SNF multiprotein complex), OG12 (transcription factor, TF GTE 12), and OG13 (ATPase family-AAA domain containing protein). The paralog groups contained Brd-members with transcription factor functions viz. PG1 (TF GTE3, *A. thaliana*), PG2 (TF GTE2, *A. thaliana*) and PG3 (TF GTE7, *O. sativa*) (**Figure 1C**). The results showed that duplication mediated *Brd-*gene copy number expansion was restricted to certain OG/PG groups in both the species (*A. thaliana*: OG1-3 and PG1-2 and *O. sativa*: OG5, PG3) (**Figure 1C**).

### 3.3 Heterogeneity of AtBRD and OsBRD-members: Impact of duplication and splicing events

**Figure 2:**
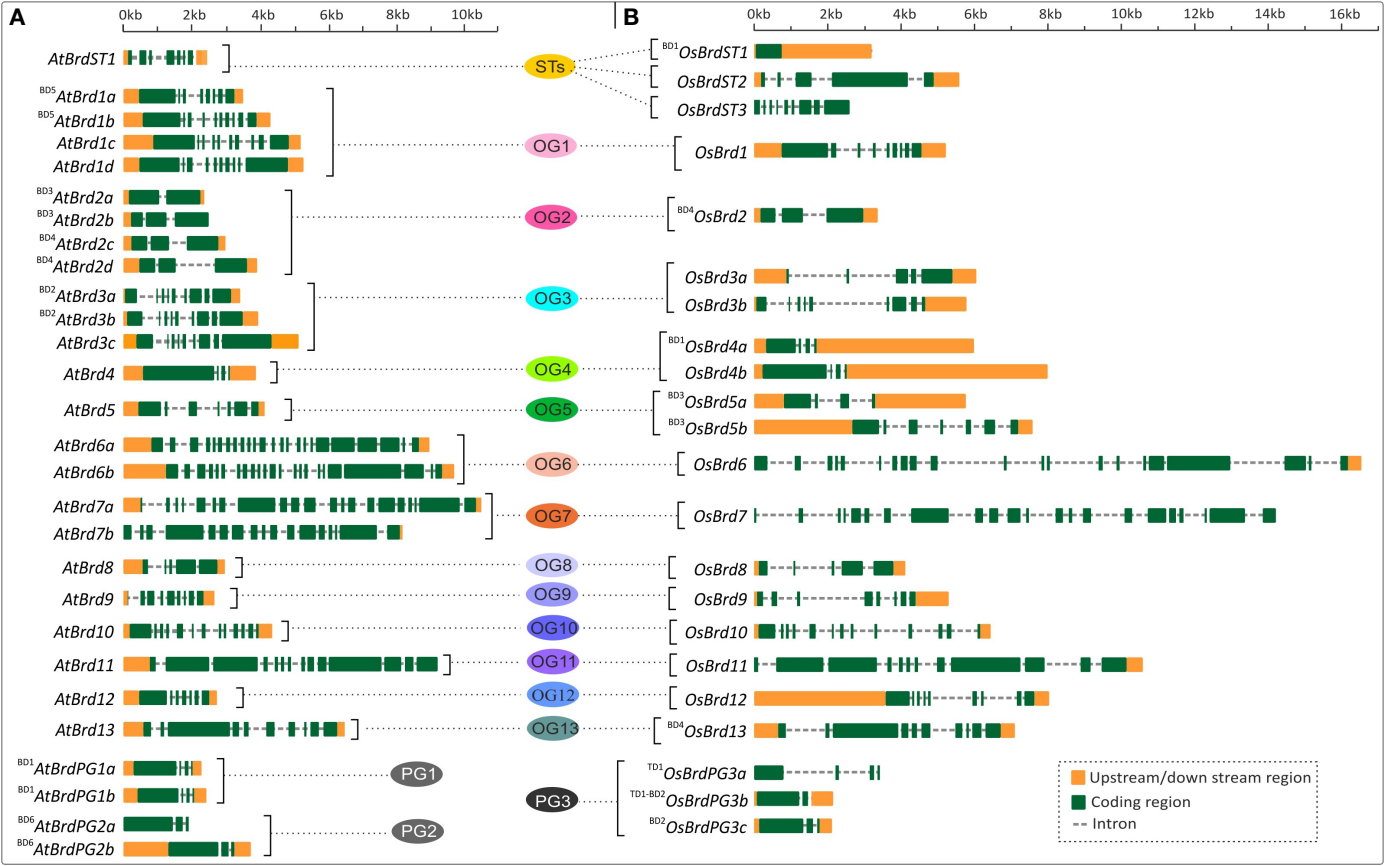
Comparison of gene structure and organization of *Brd*-genes from *A. thaliana* **(A)** and *O. sativa* **(B)**, belonging to thirteen ortholog groups (OG1-13), three paralog groups (PG1-3), and singleton category (STs). The BRD-members specific to each group are arranged side-by-side for comparison, and the designations ‘BD’ and ‘TD’ in the names indicate the block or tandem duplication. Different regions of genes are indicated by color codes (orange: upstream/downstream region including UTR, green: exons, and dashed line: intron). Scale on the top indicates the length in kilobase (kb).

The AtBrd-members showed considerable heterogeneity in length of the gene (1,919 - 10,519 bp), coding region (1,110 - 6,579 bp) and encoded proteins (369 - 2,192 aa) (**Table 1**), while the OsBrd-members displayed relatively higher variability (gene: 2,119 - 16,548 bp; coding region: 717 - 6,603 bp; protein: 238 - 2,200 aa) (**Table 2**), which was attributed to the length and number of exons, introns and 5′-/3′-UTRs. The Brd-members in most OGs/PGs showed similarity in length and exon-intron organization in the two species. For example, OG2 members harbored 2-3 exons, while OG6 and OG7 contained extremely long *Brd-*genes with 17-24 exons (**Figure 2**).

Duplication events resulted in the diversity of the Brd-genes in both *A. thaliana* and *O. sativa*. The *AtBrd*-genes originated due to six block duplication events (BD1-BD6) displayed variations in length and organization of exons, introns and UTRs (**Table 1** and **Figure 2A**). The five duplicated *OsBrd*-gene pairs due to one tandem and four block events displayed relatively higher heterogeneity than *AtBrds* (**Table 2** and **Figure 2B**). The Brd-members specific to certain OGs displayed species-specific duplication events viz. *AtBrd*-genes in OG1, OG2, and OG3 and *OsBrds* in OG5. Higher heterogeneity in gene structure was observed among the *OsBrd* paralogs (PG3) than *AtBrd* paralogs (PG1, PG2) (**Figures 2A, B**).

In addition, AS-events also affected several *Brd*-genes (~60% *AtBrds* and ~41% *OsBrds*) in different OGs/PGs. In five ortholog groups (OG1, OG4, OG5, OG8, OG9), the *Brd*-genes of both the species showed AS, however the effects of events (on UTR/exon), and number of isoforms differed, with *OsBrd1* (OG1), *AtBrd5* (OG5) displaying highest number of transcripts (**Supplementary Figure 1**). In five OGs (OG2, OG3, OG6, OG7, OG11) AS-events were evident only among *AtBrd*-genes including *AtBrd6a* (OG6) and *AtBrd11* (OG11) with maximum six isoforms, whereas in the OG12, only *OsBrd*-gene displayed AS-events (**Supplementary Figure 1**). One of the duplicated Brd-gene in PG1 (^BD1^ *AtBrdPG1b, A. thaliana*), PG3 (^BD2^ *OsBrdPG3c*, *O. sativa*) and *A. thaliana*-specific singleton *AtBrdST1* also accumulated variations to generate AS-transcripts. The AS-events affected UTRs in five genes (*AtBrd3c, AtBrd4*, *AtBrd6b*; ^BD3^ *OsBrd5a*, ^BD3^ *OsBrd5b*), and both UTR and coding regions in most of the genes (e.g., ^BD5^ *AtBrd1a*, *AtBrd1c*, *AtBrd1d*, ^BD3^ *AtBrd2b*, *AtBrd5*, *AtBrd7b*, *AtBrd8*, *AtBrd9*, *AtBrd11*, ^BD1^ *AtBrdPG1b*, *AtBrdST1*; *OsBrd1*, ^BD1^ *OsBrd4a*, *OsBrd4b*, *OsBRD8*, *OsBrd9*, *OsBrd12*, ^BD2^ *OsBrdPG3c*) (**Supplementary Figure 1**). Interestingly, among the *Brd*-gene pairs affected by certain duplication events (*A. thaliana*: BD1, BD2, BD3 and *O. sativa*: BD1, BD2), only one of the copies displayed AS, whereas both the *Brd* copies generated by BD5 (*A. thaliana*) and BD3 (*O. sativa*) were affected by AS-events (**Supplementary Figure 1**). These results show that the OG-specific Brd-members and the duplicated *Brd*-gene pairs seem to have evolved towards differential splicing patterns.

### 3.4 BRD-proteins showed heterogeneity in domain and motif organization

**Figure 3:**
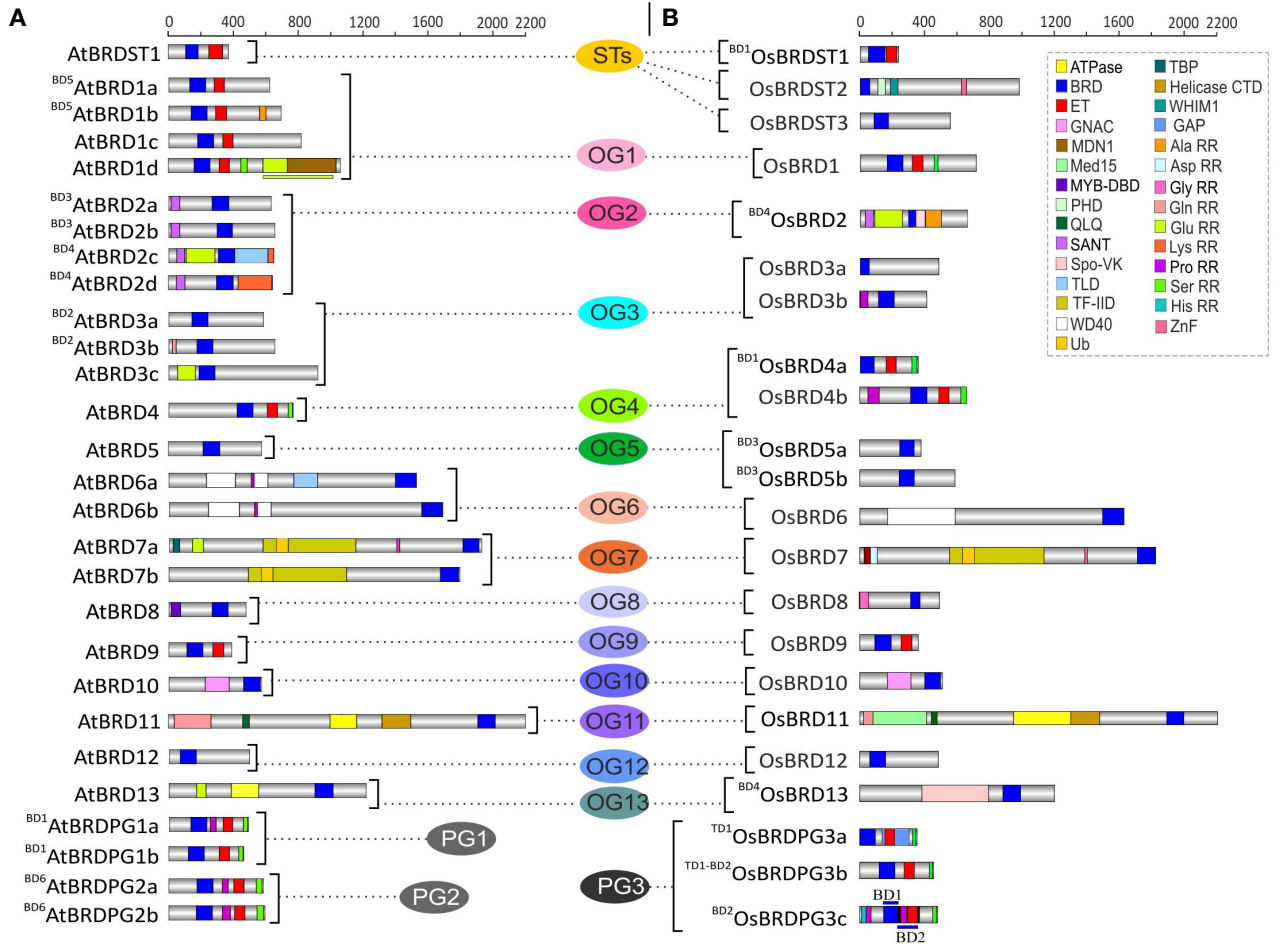
Comparison of domain architecture of BRD-proteins of *A. thaliana* **(A)** and *O. sativa* **(B)** belonging to thirteen ortholog groups (OG1-13), three paralog groups (PG1-3), and singleton category (STs). The BRD-members specific to each group are arranged side-by-side for comparison, and the designations ‘BD’ and ‘TD’ in the names indicate the block or tandem duplication. Domains/important functional sites (CDD and PROSITE prediction) are shown by different color codes. In AtBRD1d, OsBRDPG3c, color-coded lines (above/below) indicate spread of domains to adjacent regions. Scale on the top indicates the protein length (number of amino acids).

Apart from bromodomain (BRD), the AtBRD and OsBRD proteins harbored more than 25 other domains, including the most prominent extra-terminal (ET) domain (**Figure 3**). Certain domains were specific to AtBRDs (e.g. TLD, MDN1, Lys rich repeats, **Figure 3A**) and OsBRDs (e.g. PHD, WHIM1, Spo-VK, Med15, Asp/His rich repeats, **Figure 3B**). Broadly, AtBRD and OsBRD-members were divided into four types, a) containing only BRD, b) BRD + ET, c) BRD + other domains (other than ET), and d) BRD + ET + other domains. Notably, certain OGs with single BRD-members (OG9, OG10, OG12) showed conserved domain architecture between *A. thaliana* and *O. sativa* members, whereas, other OGs with single (OG8, OG11, OG13) and multiple BRD-members (OG1-OG7), including duplicated-BRDs displayed domain differences between the two species (**Figure 3**). AtBRDs specific to *A. thaliana* PG1 and PG2 showed minor variations, whereas PG3-specific *(O. sativa*) member, ^BD2^OsBRDPG3c (duplicated by BD2 event) displayed a dual-BRD domain architecture (2^nd^ BRD partially overlapped with the ET domain) (**Figure 3B**). Furthermore, among the BRDs of the two species 15 conserved motifs (M1 - M15) were identified, of which M1 and M2 were most prevalent, and the duplicated members in the two species showed conserved signatures (**Supplementary Tables 1**, **2** and **Supplementary Figure 2**).

### 3.5 Duplications and AS-events affected domain architecture of BRDs

**Figure 4:**
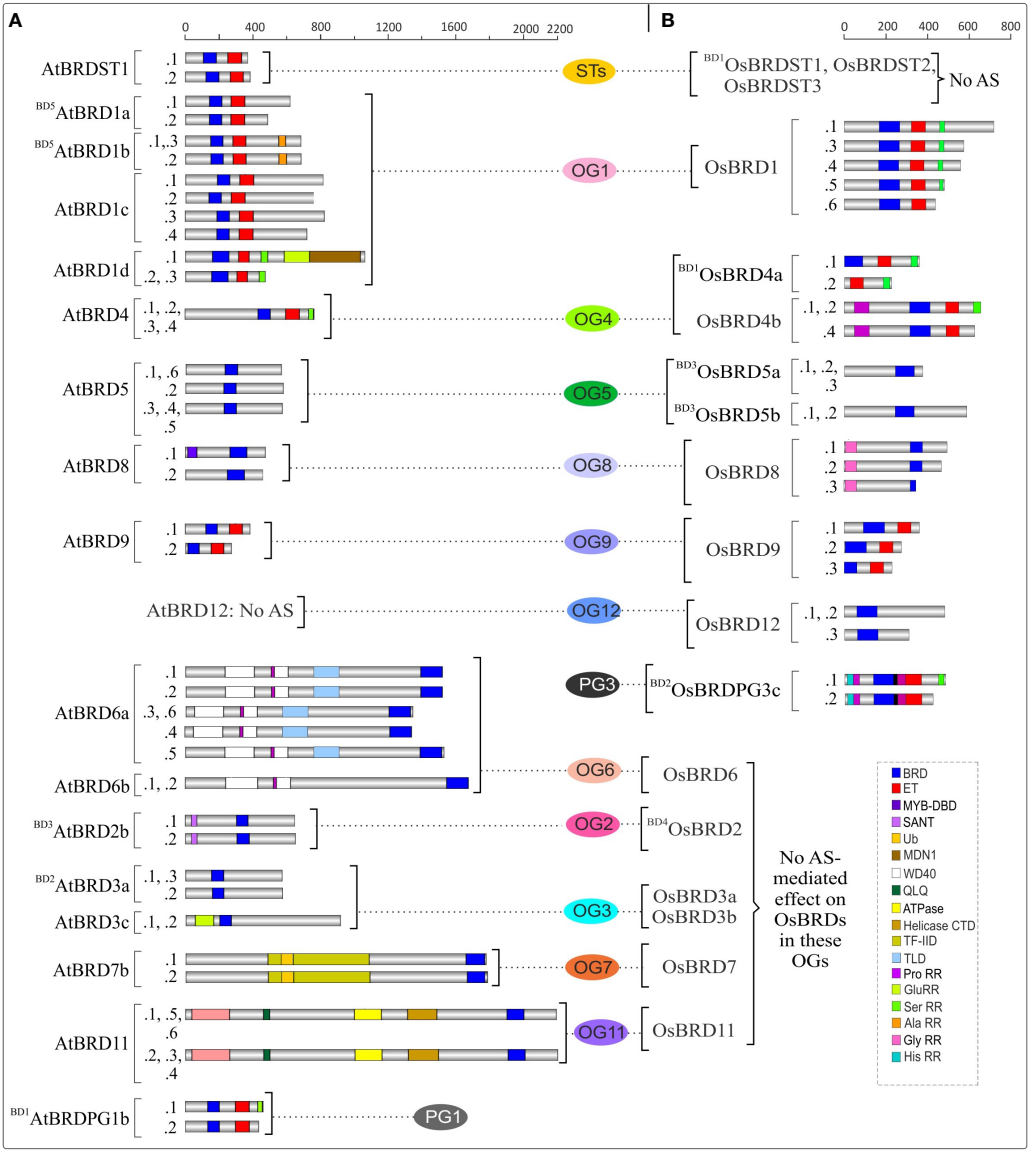
Alternative splicing (AS)-mediated changes in domain architecture of BRD isoforms of *A. thaliana* **(A)** and *O. sativa* **(B)** belonging to certain ortholog groups (OGs), paralog groups (PGs), and singleton category (STs). The members specific to each group are arranged side-by-side for comparison, and the designations ‘BD’ and ‘TD’ in the names indicate the block or tandem duplication. Domains/important functional sites among different isoforms (constitutive:.1 and alternative: .2 to .6) are shown by different color codes. Scale on the top indicates the protein length (number of amino acids).

Duplication events affected the domain architecture of duplicate AtBRD and OsBRD-pairs. Four of the six block events (BD1, BD2, BD4, BD5) resulted in domain variations among the members of AtBRD-pairs compared to BD3 and BD6 events (**Figure 3A**). Among the OsBRD-duplicates, domain diversity was seen among members originated by tandem (TD1) and three block duplications (BD1, BD2, BD4), with substantial heterogeneity in BD2 and BD4 generated pairs (**Figure 3B**). Coding-region (exon) specific AS-events also affected the domain diversity of several AtBRD and OsBRD-members (**Figure 4**). Eight AtBRDs from OG1, OG6, OG8-9, PG1 and ST1 displayed AS-mediated loss of certain domains (MYB-DBD: AtBRD8.2; MDN1: AtBRD1d.2, 1d.3; Ser RR: ^BD1^AtBRDPG1b.2) or N/C-terminal region (^BD5^AtBRD1a.2, AtBRD1c.2, 1c.4; AtBRD1d.2, 1d.3; AtBRD9.2; AtBRD6a.3, 6a.4, 6a.6; AtBRDST1.1; ^BD1^AtBRDPG1b.2) among the alternative isoforms (**Figure 4A**). Likewise, eight OsBRDs displayed AS-mediated loss of BRD (complete: ^BD1^OsBRD4a.2; partial: OsBRD8.2; OsBRD9.3), Ser RR (OsBRD1.6; OsBRD4b.3; OsBRDPG3c.2), and C-terminal truncation (OsBRD1.3, 1.4, 1.5, 1.6; OsBRD8.2, 8.3; OsBRD12.3; OsBRDPG3c.2) (**Figure 4B**). The AS-mediated loss/truncation of BRD-region was specific to three OsBRDs, and not observed among AtBRDs. Both duplications and AS-events enhanced the diversity of BRD-members in two species.

### 3.6 *Cis*-elements indicates responsiveness of *Brd*-genes to diverse intrinsic and extrinsic factors

**Figure 5:**
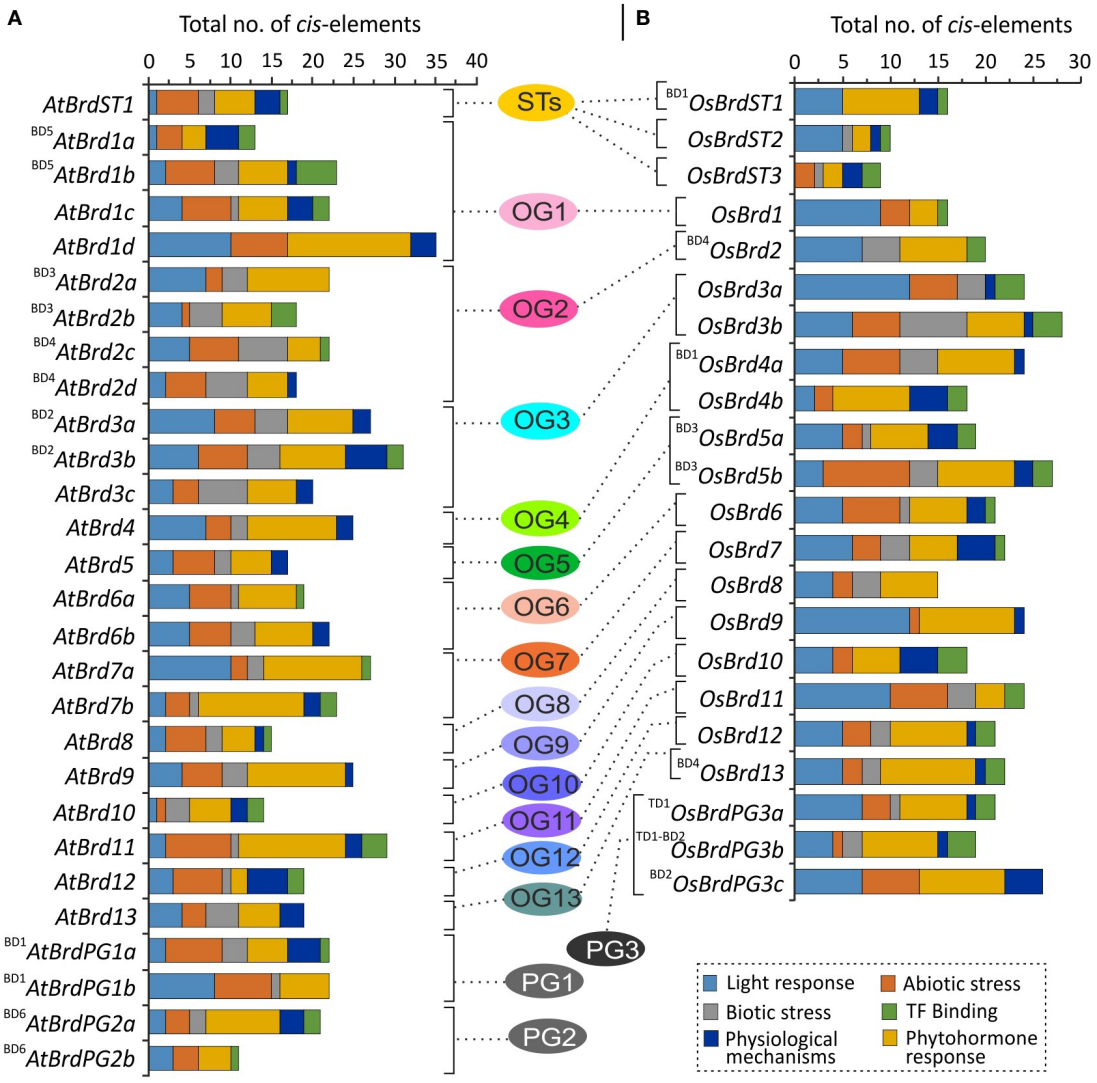
Diversity of *cis*-regulatory elements in the upstream region (-2000 bp) of *A. thaliana* **(A)** and *O. sativa* **(B)** *Brd*-genes, belonging to thirteen ortholog groups (OG1-13), three paralog groups (PG1-3), and singleton category (STs), as per analysis at PlantCARE database. The Brd-members specific to each group are arranged side-by-side for comparison, and the designations ‘BD’ and ‘TD’ in the names indicate the block or tandem duplication. Different functional categories of *cis*-motifs are shown by different color code.

The upstream regions of *Brd*-genes in both species contained *cis-*elements associated with diverse functions, including response to light, stress conditions (abiotic: low temperature, anaerobic condition; biotic: wound, defense, elicitor-mediated activation), phytohormones (abscisic acid, auxin, salicylic acid, jasmonic acid, ethylene, gibberellin), and some physiological functions (**Supplementary Figures 3**, **4**). While certain *Brd*-genes contained higher number of motifs for biotic stress (^BD4^ *AtBrd2c*, ^BD4^ *AtBrd2d*, *AtBrd3c*; ^BD4^ *OsBrd2*, *OsBrd3b*, ^BD1^ *OsBrd4a*, *OsBrd8*) and physiological functions (*AtBrdST1*, ^BD5^ *AtBrd1b*, *AtBrd1c-1d*, ^BD3^ *AtBrd2b*, ^BD2^ *AtBrd3b*, *AtBrd12*, ^BD1^ *AtBrdPG1a*, ^BD6^ *AtBrdPG2a*; *OsBrd4b*, ^BD3^ *OsBrd5a*, *OsBrd7*, *OSBrd10*, ^BD2^ *OsBrdPG3c*), few lacked response motifs for phytohormone (*OsBrd3a*), abiotic stress (^BD1^ *OsBrdST1*, *OsBrdST2*, ^BD4^ *OsBrd2*) and light (*OsBrdST3*) (**Figure 5**). Some *cis-*elements were specific to certain *Brd*-genes viz. NON-box (*OsBrd4b*), motif1 (*OsBrd7*), and TATC-box (*OsBrd12*, ^BD3^ *OsBrd5b*), MBSI (^TD1^ *OsBrdPG3a* in *O. sativa*) (**Supplementary Figures 3**, **4**). Diversity of *cis-*elements indicate responsiveness of *Brd*-members towards diverse stimuli, and showed almost no conservation among different OGs/PGs (**Figures 5A, B**).

### 3.7 Duplication events affected the *cis*-element diversity and promoter structure

**Figure 6:**
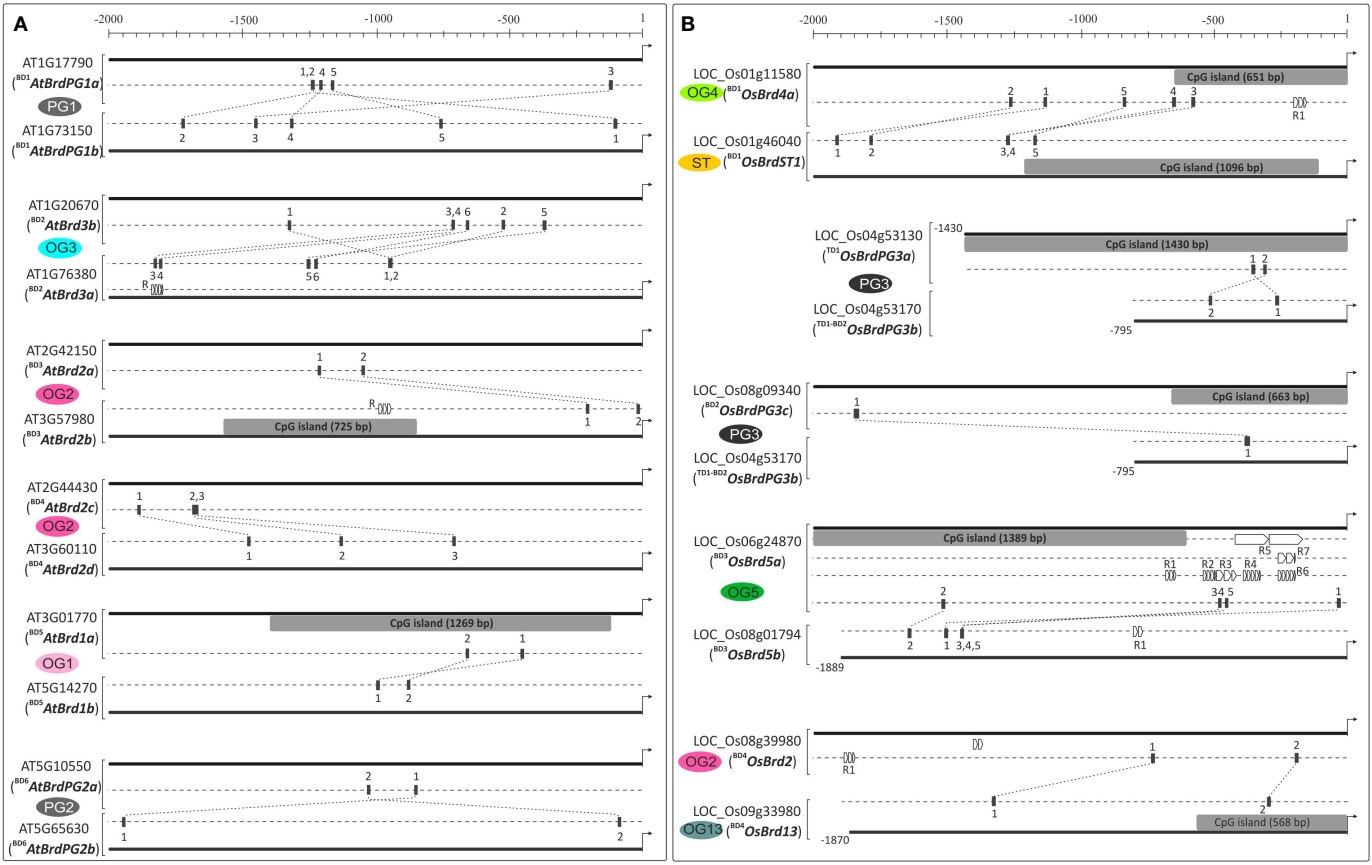
Analysis of upstream region (-2000 bp) of duplicated *Brd*-genes of *A. thaliana* **(A)** and *O. sativa* **(B)** on PlantPAN3.0 database, for difference in CpG islands (grey boxes), transcription factor binding sites (TFBS, indicated with numerals 1-6 in different genes) and repetitive motifs (R). The designation (and locus number) of Brd-members, association with ortholog group (OG)/paralog group (PG)/singleton category (ST) is indicated, and the designations ‘BD’ and ‘TD’ in the names indicate block or tandem duplication. Scale on the top indicates the length of upstream region (bp), arrow towards right indicates translation start site.

The tandem and block duplications affected the *cis-*element diversity among duplicated *Brd*-gene pairs in both the species. For example, *Brd*-gene pair ^BD5^ *AtBrd1a-* ^BD5^ *AtBrd1b* (OG1) differed in *cis*-elements for light, abiotic and biotic stress (wound, defense), phytohormones (gibberellin, jasmonic acid) and physiological functions (meristem and endosperm-specific expression, circadian control), while ^BD1^ *AtBrdPG1a*-^BD1^ *AtBrdPG1b* (PG1) differed in elements for light, defense, abiotic stress, phytohormones (ethylene, gibberellin), TF-binding and physiological functions (**Figure 5A** and **Supplementary Figure 3**). Similarly, in *O. sativa* ^BD1^ *OsBrd4a*-^BD1^ *OsBrdST1* pair (OG4, ST) differed in *cis*-element copy numbers, and ^BD1^ *OsBrdST1* also lacked elements for abiotic and biotic stress. Also, OG5-specific ^BD3^ *OsBrd5a*-^BD3^ *OsBrd5b* displayed differences in elements for light, abiotic and biotic stresses, and certain physiological mechanisms (**Figure 5B** and **Supplementary Figure 4**). The duplications did not affected the length of promoter region of *AtBrd*-gene pairs, however substantial length differences were evident among most of the duplicate *OsBrd*-pairs (**Figure 6**). In addition, duplicated *AtBrd* and *OsBrd*-pairs displayed differences in the arrangement of TFBS (all duplicate pairs), repeat motifs (^BD2^ *AtBrd3a*-^BD2^ *AtBrd3b*; ^BD3^ *AtBrd2a*-^BD3^ *AtBrd2b*; ^BD1^ *OsBrd4a*-^BD1^ *OsBrdST1*, ^BD3^ *OsBrd5a*-^BD3^ *OsBrd5b*, ^BD4^ *OsBrd2*-^BD4^ *OsBrd13*), and CpG islands (^BD3^ *AtBrd2a*-^BD3^ *AtBrd2b*; ^BD5^ *AtBrd1a*-^BD5^ *AtBrd1b*; All *OsBrd*-pairs) (**Figure 6**). Overall, the duplication event seems to have affected the promoters of the Brd-members, which might be important for their responsiveness towards diverse intrinsic/extrinsic factors.

### 3.8 *AtBrd* and *OsBrd*-genes showed tissue-and stress-specific expression differences

**Figure 7:**
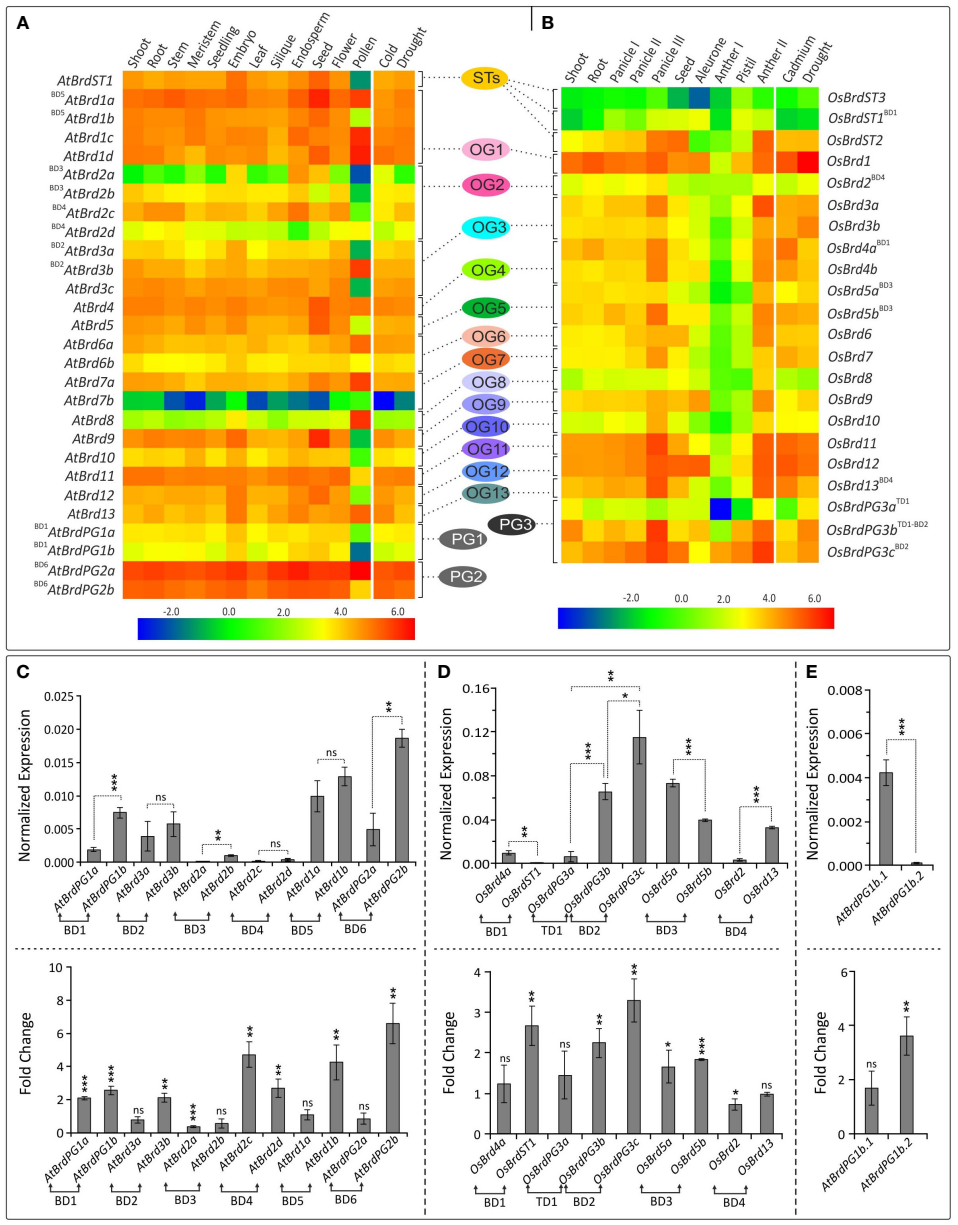
Expression analysis of *Brd*-genes: Heatmap-based analysis of RNA-Seq data for tissue-specific and abiotic stress-responsive expression pattern of *Brd*-genes of *A. thaliana* **(A)** and *O. sativa* **(B)**, belonging to thirteen ortholog groups (OG1-13), three paralog groups (PG1-3), and singleton category (STs). Different tissues and stress conditions are indicated on the top, and names of *Brd*-genes are shown on the sides, with designations ‘BD’ and ‘TD’ indicating block or tandem duplication event. A continuous color gradient scale is indicative of the expression level (blue: low levels; red: high). RT-qPCR analysis of transcript levels of six duplicated *AtBrd*-gene pairs **(C)** and five *OsBrd*-gene pairs **(D)** in seedling tissues (top panels), and in response to salt stress (NaCl, 150 mM, bottom panels), using reference genes (*AtActin*; *OselF1α*). Designations ‘BD’ and ‘TD’ indicate block or tandem duplication event. **(E)** Expression pattern of two isoforms of *AtBrdPG1b* (constitutive:.1; alternative:.2) in Arabidopsis seedlings (top panel), and in response to salt stress (bottom panel). The analysis was carried out in triplicate, data is represented as mean ± SD, and statistical significance is indicated by *(*p <*0.05), **(*p <*0.01), ***(*p <*0.001), ns (no significant difference).

The analysis of RNA-Seq data showed tissue- and stress-specific abundance patterns of *AtBrd*-genes. In general, *AtBrd*s from OG8, OG7 (*AtBrd7b*), OG2 (two genes: ^BD3^ *AtBrd2a*, ^BD4^ *AtBrd2d*) and PG1 showed lower transcript levels compared to Brd-members from PG2, OG1, OG3-4, OG9, OG11 and PG3. Genes ^BD5^ *AtBrd1a* and ^BD6^ *AtBrdPG2a* displayed high transcript levels in most tissues, while *AtBrd7b* showed the lowest (**Figure 7A**). Pollen tissue displayed abundance of seven *AtBrds* (^BD5^ *AtBrd1a*, *AtBrd1c*, *AtBrd1d*, ^BD2^ *AtBrd3b*, *AtBrd7a*, *AtBrd8*, ^BD6^ *AtBrdPG2a*), while many others showed lowest levels. Substantial tissue-specific differences were observed among the members of two duplicate pairs, ^BD3^ *AtBrd2a*-^BD3^ *AtBrd2b* and ^BD4^ *AtBrd2c*-^BD4^ *AtBrd2d* (**Figure 7A**). In response to cold and drought, most *AtBrds* showed up-regulation, with higher levels observed for ST1, OG1 and PG2 Brd-members compared to *AtBrd7b* (down-regulated), while *AtBrd2a* and *AtBrd8* showed weak response (**Figure 7A**). Certain *AtBrds* (*AtBrd2a*, *AtBrd2c*, *AtBrd2d*, *AtBrd12, AtBrd13*) showed variation in the response to stresses.

In *O. sativa*, *OsBrds* from clusters OG8, OG10 and PG3 (^TD1^ *OsBrdPG3a*) and two STs (^BD1^ *OsBrdST1, OsBrdST3*) showed low transcript levels, while members from OG1, OG11-12 and PG3 (^BD2^ *OsBrdPG3c*) were abundant in most tissues. In general, the *OsBrds* showed low levels in anther I tissue and highest in panicle II and anther II (**Figure 7B**). Most *OsBrd*-duplicates displayed tissue-specific differences, with maximum variation in ^BD1^ *OsBrd4a*-^BD1^ *OsBrdST1* and ^BD4^ *OsBrd2*-^BD4^ *OsBrd13* pairs (**Figure 7B**). The *OsBrds* also responds variably to cadmium and drought stress conditions, with two members from ST (*OsBrdST3*, ^BD1^ *OsBrdST1*) and *OsBrd8* showing lower response compared to strong upregulation of *OsBrd1*, *OsBrd11* and *OsBrd12* (**Figure 7B**). Also, ^BD4^ *OsBrd2*, ^TD1^ *OsBrdPG3a*, ^BD3^ *OsBrd5a* and ^TD1-BD2^ *OsBrdPG3b* showed different response or trend in two conditions (**Figure 7B**).

The RT-qPCR analysis of duplicate *Brd*-pairs (*AtBrd*: 6-pairs; *OsBrds*: 5 pairs) in seedlings tissue showed difference in basal transcript levels and response to salinity. Among the *AtBrd*-duplicates, ^BD1^ *AtBrdPG1b*, ^BD3^ *AtBrd2b*, and ^BD6^ *AtBrdPG2b* showed higher transcript levels than corresponding duplicate members, while the Brd-members from BD2, BD3 and BD4-duplicate groups showed comparable levels (**Figure 7C**, top panel). In response to salt stress, seven *AtBrds* (^BD1^ *AtBrdPG1a*; ^BD1^ *AtBrdPG1b*, ^BD2^ *AtBrd3b*, ^BD4^ *AtBbrd2c*, ^BD4^ *AtBrd2d*, ^BD5^ *AtBrd1b*, ^BD6^ *AtBrdPG2b*) were up-regulated (~2-6-fold), *AtBrd2a* was down-regulated and four Brd-members (^BD2^ *AtBrd3a*, ^BD6^ *AtBrdPG2a*, ^BD3^ *AtBrd2b*, ^BD5^ *AtBrd1a*) remained unaffected (**Figure 7C**, bottom panel). In rice seedlings among the *OsBrd*-pairs, ^BD1^ *OsBrd4a*, ^BD4^ *OsBrd13*, ^BD3^ *OsBrd5a*, ^BD2^ *OsBrdPG3c* showed relatively higher basal transcript levels than the duplicate member (**Figure 7D**, top panel). Under salt stress, five *OsBrds* were up-regulated (^BD1^ *OsBrdST1*, ^BD3^ *OsBrd5a*, ^BD3^ *OsBrd5b*, ^TD1-BD2^ *OsBrdPG3b*, ^BD2^ *OsBrdPG3c*), ^BD4^ *OsBrd2* was down-regulated, and three (^BD1^ *OsBrd4a*, ^BD4^ *OsBrd13*, ^TD1^ *OsBrdPG3a*) showed no significant change (**Figure 7D**, bottom panel). In most of the duplicate Brd-pairs in the two species, one of Brd-members showed response to salinity. Further, analysis of alternative splicing of *AtBrdPG1b*-gene by RT-qPCR assay (using splice variant-specific primers), showed differential basal levels of constitutive (*AtBrdPG1b.1*) and alternative (*AtBrdPG1b.2*) transcripts (**Figure 7E**, top panel). However, the splicing pattern of alternative transcript (*AtBrdPG1b.2*) was modulated in response to salinity (**Figure 7E**, bottom panel). Collectively, these results show that the *Brd*-duplicates have evolved for differential response towards intrinsic/extrinsic factors.

### 3.9 Sequence divergence and key conserved sites in bromodomain (BRD) region of BRD-homologs

**Figure 8:**
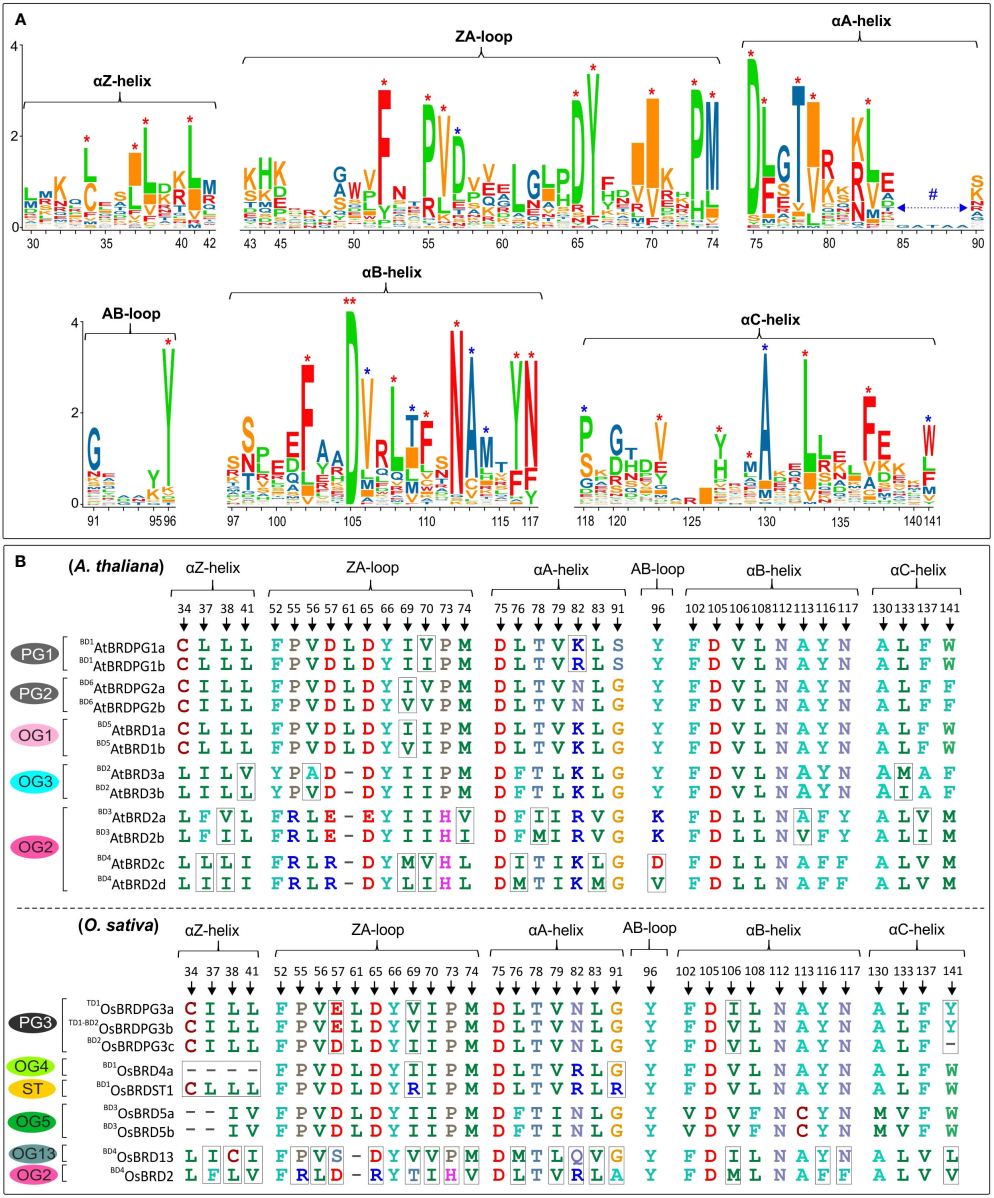
**(A)** Sequence logo analysis of the bromodomain (BRD)-region of 28 AtBRD and 22 OsBRD-homologs, indicating conserved residues in the key BRD-fold elements (helices: αZ, αA, αB, αC; loops: ZA, AB). ‘*’ indicates conserved sites (red ‘*’: conserved residues also in human BRDs; blue ‘*’: conserved sites in At- and OsBRDs), and ‘**’ indicates an invariant residue, and # indicates region specific to a single OsBrd (OsBRD2). Positions of amino acid residues (as per the alignment in **Supplementary Figure 5**) are indicated on the x-axis. **(B)** Comparison of conserved sites in key elements of BRD-fold among the duplicated BRDs of *A. thaliana* (top panel) and *O. sativa* (bottom panel). The association of duplicate-BRDs to different OGs/PGs or ST category is indicated, designations ‘BD’ and ‘TD’ indicate type of duplication event, while the variations at key positions are indicated by rectangular boxes.

The bromodomain (BRD) region showed more length variation among OsBRDs (range: 57-133 aa) than AtBRDs (range: 94-133 aa), particularly due to two OsBRDs (OsBRD3a, OsBRDST2), which harbored long N-terminal deletion leading to exceptionally small BRD-regions (**Supplementary Figure 5**). Also, three-pairs of AtBRDs (^BD5^AtBRD1a-^BD5^AtBRD1b; ^BD3^AtBRD2a-^BD3^AtBRD2b; ^BD4^AtBRD2c-^BD4^AtBRD2d); and OsBRDs (^BD1^OsBRD4a-^BD1^OsBRDST1, ^TD1-BD2^OsBRDPG3b-^BD2^OsBRDPG3c; ^BD4^OsBRD2-^BD4^OsBRD13) showed indel variations (**Supplementary Figure 5**). Several conserved residues, similar to human BRDs, were identified in the characteristic BRD-fold elements viz. αZ-helix (Leu-34, Ile/Leu-37, Leu-38, Leu/Ile-41), ZA-loop (Phe-52; Pro-55, -73; Val-56; Asp-65; Tyr-66; Ile-70; Met-74), αA-helix (highly conserved Asp-75; Leu-76, -83; Thr-78; Ile-79), small AB-loop (conserved Tyr-96), αB-helix (invariant Asp-105; Phe-102, -110; Leu-108; Asn-112, -117; Tyr-116), and αC-helix (Val-123; Tyr-127; Met-129; Leu-133; Phe-137). Plant-specific signatures were also evident in ZA loop (Asp-57), αB-helix (Val-106, Thr-109, Ala-113, Met-114) and αC (Pro-118, Ala-130, Trp-141) (**Figure 8A**). Most of these key sites were conserved among the BRD-duplicates, however AtBRD-pairs displayed variations at one (Ile/Val, in ZA-loop, OG1, PG2) to seven sites (^BD4^AtBRD2c-^BD4^AtBRD2d, OG2) (**Figure 8B**, top panel). The OsBRD-pairs showed more heterogeneity with up to 20 variable sites (^BD4^OsBRD2-^BD4^OsBRD13, OG2, OG13), and loss of αZ-helix in ^BD1^OsBRD1a (BD1 pair: ^BD1^OsBRD4a-^BD1^OsBRDST1) (**Figure 8B**, bottom panel). Such variations can alter the interactions of the BRD-fold with chromatin.

**Figure 9:**
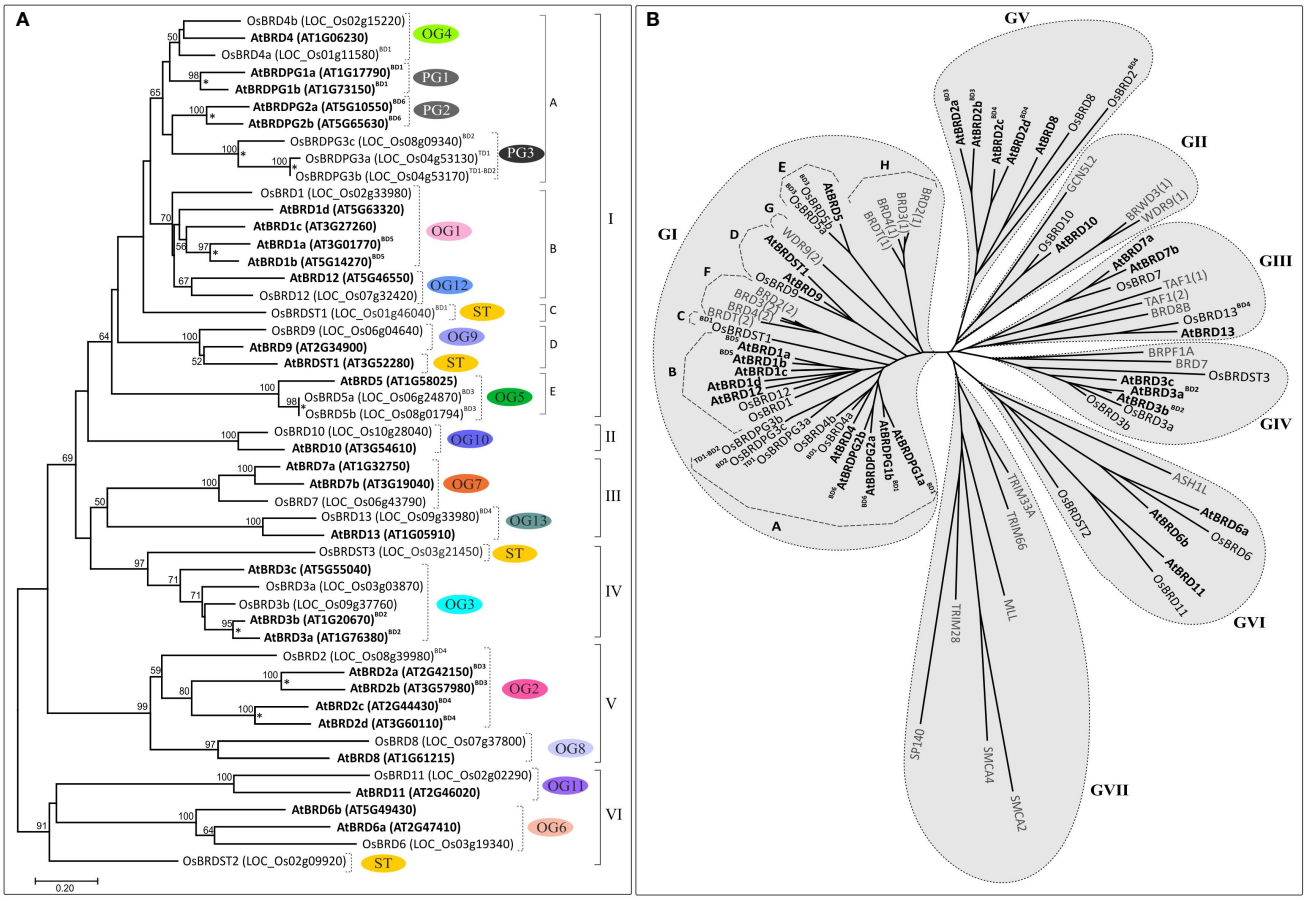
**(A)** Neighbor-joining phylogenetic tree based on the bromodomain (BRD)-regions of *A. thaliana* and *O. sativa* Brd*-*homologs (including the block and tandem duplicates) generated by MEGA-X software. Major clusters are indicated by Roman numerals (I-VI), while sub-clusters are shown by letters (A-E). The ortholog group (OG), paralog group (PG) or singleton category (ST) designation is also indicated. Numbers at the nodes indicate bootstrap values (in %, for 500-replicates), and taxa names (AtBRDs: bold font, OsBRDs: regular font) include Brd-designation used, locus numbers (in parenthesis), and type of duplication events (BD: block duplication; TD: tandem duplication). ‘*’ Indicate the gene duplication event in the species. **(B)** Radiation tree of the BRD-regions of *A. thaliana* (AtBRDs: bold font style), *O*. *sativa* (OsBRDs: regular font style), and some representative human BRD-homologs (BRD2-4, BRD8B, BRDT, WDR9, TAF1, BRWD3, BRPF1A, BRD7, GCN5L2, ASH1L, TRIM33A, TRIM66, MLL, SMCA2, SMCA4, TRIM28, SP140, shown in grey font style), placed into seven major groups (GI-GVII) and subgroups (A-H). Designation ‘BD’ and ‘TD’ indicated block and tandem duplication events, while numerals in parenthesis (1/2) indicate two different domains of the dual-BRD-containing homologs.

Cluster analysis based on BRD-region placed the 50 BRD sequences from two species into six clusters (I - VI) (**Figure 9A**). The intra-group site variability ranged from 21% to 67.3% (II and VI), while the intergroup variability was 60.4% (I/IV) to 77.8% (III/VI). Different clusters/sub-clusters represented BRD-members specific to different OGs/PGs (**Figures 2** and **9**). Largest cluster I was divided into five sub-clusters: IA (OG4, PG1, PG2, PG3), IB (OG1, OG12), IC (^BD1^OsBRDST1), ID (OG9, AtBRDST1), IE (OG5). Other clusters also displayed similar trend viz. II (OG10), III (OG7, OG13), IV (OG3, OsBRDST3), V (OG2, OG8), and VI (OG6, OG11, OsBRDST2). The BRD-regions of all AtBRD-duplicate pairs (events: BD1 to BD6), and three OsBRD-pairs (events: TD1, BD2, BD3) grouped together in respective clusters indicative of less divergence (**Figure 9A**). On the contrary, members of two BD-pairs (^BD1^OsBRD4a-^BD1^OsBRDST1; ^BD4^OsBRD2-^BD4^OsBRD13) were clustered differently (IA, IC and III, V) indicating high divergence (**Figure 9A**). Consistency in the BRD-based clustering and the OG/PG grouping suggest similar divergence of the domain vis-à-vis total protein. Analysis with several human single/dual BRD-regions identified clusters/sub-clusters specific to plants (GIA - IE, GV) and human sequences (GVII, IF – IH) (**Figure 9B**). Interestingly, the two domains of human dual BRD-members clustered with At/OsBRDs from different groups. For example, Bromodomain (1) sequences of BRD2-4, BRDT (sub-cluster IH) was close to IE (AtBRD5, ^BD3^OsBRD5a-^BD3^OsBRD5b), and Bromodomain (2) sequences (sub-cluster IF) were close to plant-specific sub-clusters (ID, IC, IB). On the contrary, two BRD-domains (1, 2) of human WDR9 displayed high divergence and placed in different clusters (GI-G, GII) (**Figure 9B**).

### 3.10 Heterogeneity mediated structural variations in the bromodomain (BRD)-fold

**Figure 10:**
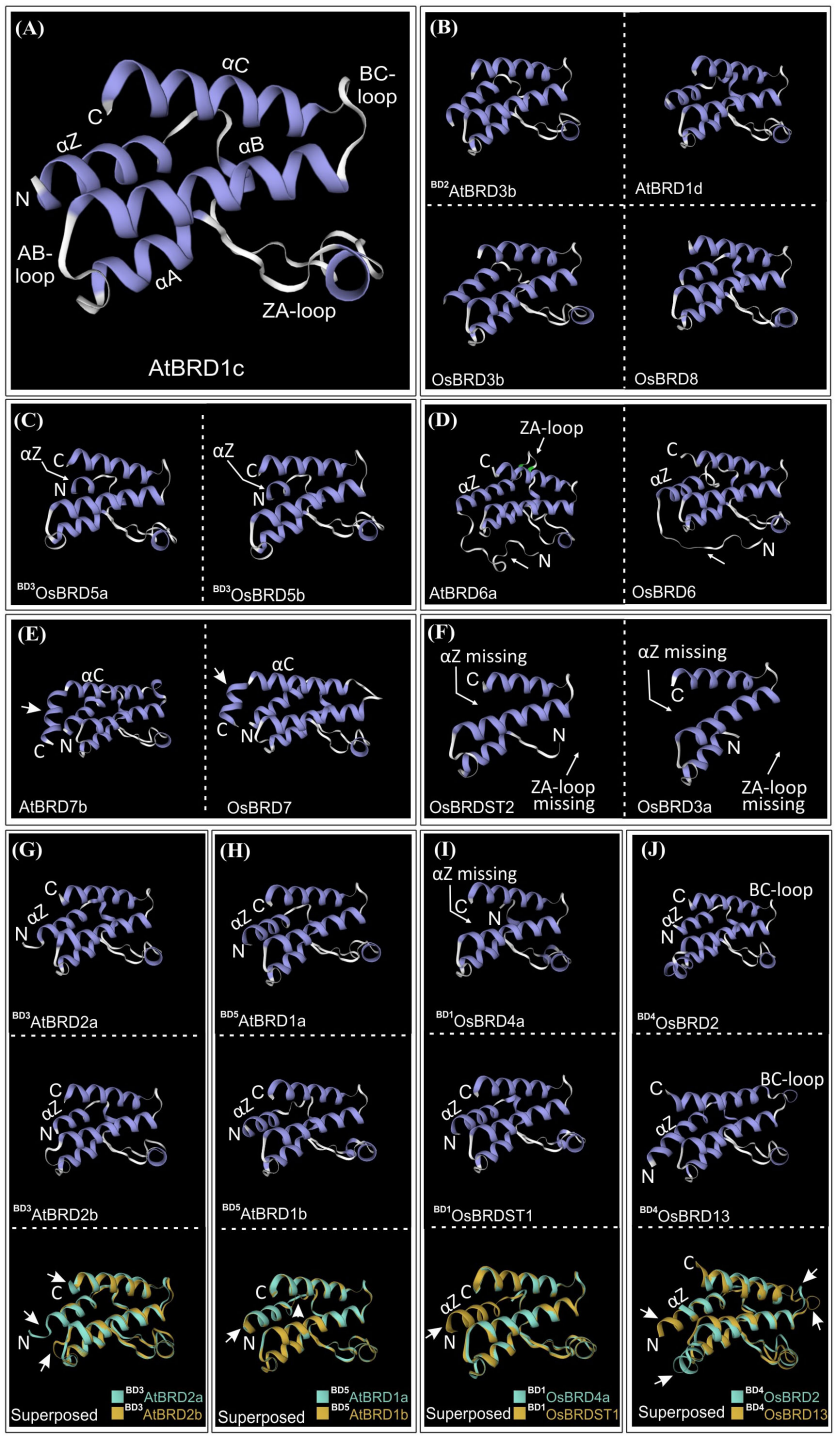
Homology models of bromodomain (BRD)-folds of *A. thaliana* and *O. sativa* Brd-homologs generated at SWISS-MODEL workspace. **(A)** AtBRD1c model showing key BRD-fold elements (α-helices: αZ, αA, αB, αC; loops: ZA, AB and BC), **(B)** ^BD2^AtBRD3b, AtBRD1d, OsBRD3b and OsBRD8, **(C)** ^BD3^OsBRD5a and ^BD3^OsBRD5b, **(D)** AtBRD6a and OsBRD6, **(E)** AtBRD7b and OsBRD7, **(F)** OsBRDST2 and OsBRD3a. Structural superposition of homology-models of duplicate BRDs (shown in different colors): **(G)** ^BD3^AtBRD2a-^BD3^AtBRD2b, **(H)** ^BD5^AtBRD1a-^BD5^AtBRD1b, **(I)** ^BD1^OsBRD4a-^BD1^OsBRDST1, and **(J)** ^BD4^OsBRD2-^BD4^OsBRD13. Structural variations (due to sequence/length heterogeneity or duplication events) are indicated by arrows.

The BRD-fold is comprised of four α-helices (αZ, αA, αB, αC) and three loops (ZA, AB, BC) (**Figure 10A**), which were affected by both length/sequence variations in At- and OsBRDs (**Supplementary Figure 5**). Conserved BRD-fold was observed for several At/OsBRDs (**Figure 10B**), however sequence divergence affected prominent structural features viz. truncated αZ-helix due to N-ter deletion (^BD3^OsBRD5a, ^BD3^OsBRD5b, **Figure 10C**), an extended region before αZ-helix (AtBRD6a, OsBRD6), variation in ZA-loop (AtBRD6a) (**Figure 10D**), an additional α-helix after αC-helix due to long C-ter region (ATBRD7b, OsBRD7, **Figure 10E**), and complete loss of αZ-helix and ZA-loop (OsBRD3a, OsBRDST2, **Figure 10F**). Superposition of duplicated-BRD homology models revealed heterogeneity in the BRD-fold, including minor structural variations in AtBRD-pairs with low divergence (^BD3^AtBRD2a-^BD3^AtBRD2b, **Figure 10G**; ^BD5^AtBRD1a-^BD5^AtBRD1b, **Figure 10H**), loss of αZ-helix in ^BD1^OsBRD4a (^BD1^OsBRD4a-^BD1^OsBRDST1, **Figure 10I**), and variations in αZ, αC and BC loop (^BD4^OsBRD2-^BD4^OsBRD13, **Figure 10J**). Such structural variations might alter the characteristics and BRD-associated functions of duplicate-members.

### 3.11 Duplication events affected the *Brd*-gene numbers among higher plants

**Figure 11:**
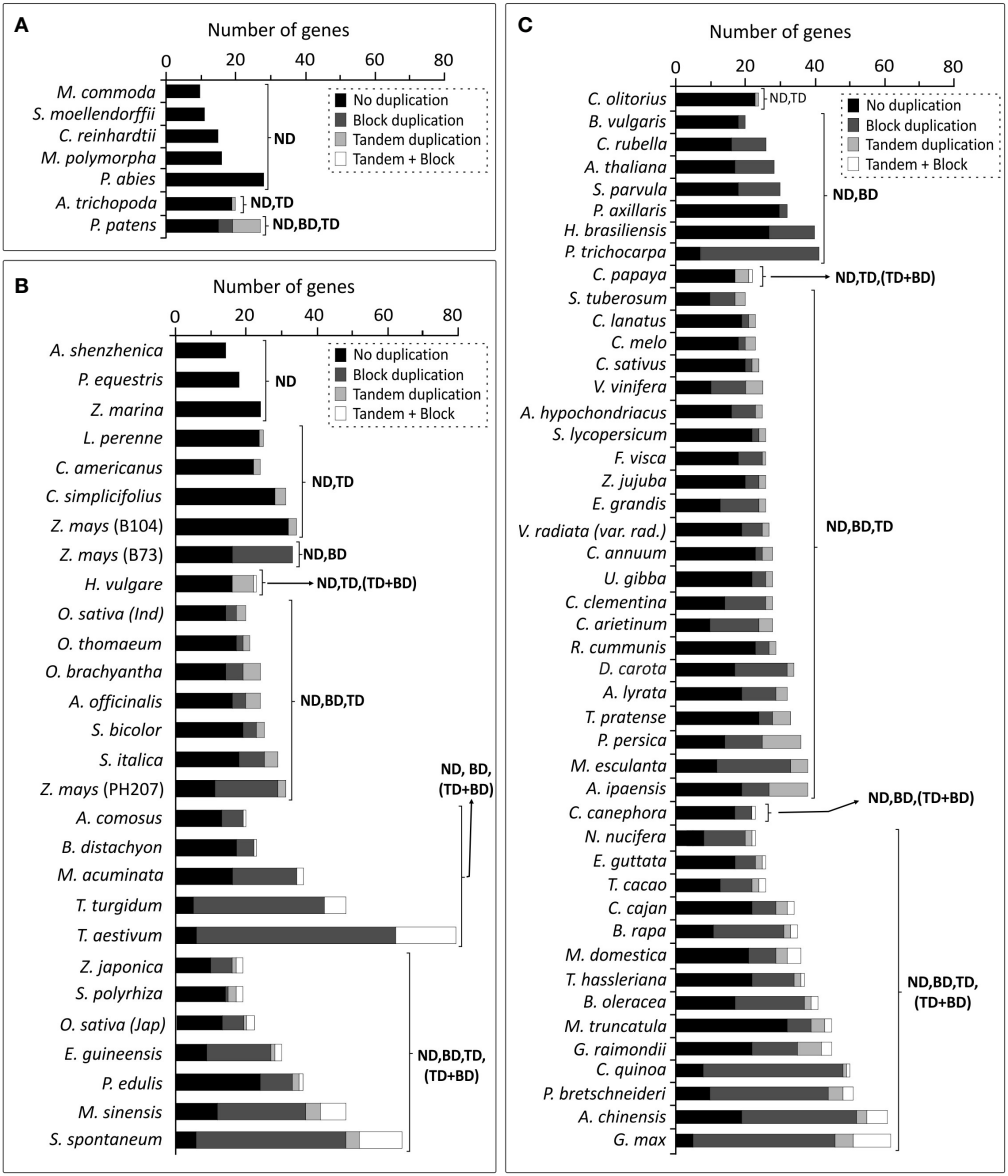
Comparative assessment of duplication events affecting *Brd*-gene copies among different plant genomes: **(A)** lower photosynthetic organisms, **(B)** monocots, and **(C)** dicots, as per analysis at PLAZA database (version 4.5). Types of duplication events are indicated by different grey shades and designations ND (no duplication), TD (tandem duplication), BD (block duplication), and TD + BD (combined tandem and block duplication).

Based on the results obtained in *A. thaliana* and *O. sativa*, the impact of duplication events was evaluated on *Brd*-genes among genomes of 79 photosynthetic organisms, including monocots and dicots. *Brd*-gene copies among four lower organisms ranged from 09-16, while *P. abies* harboured 28 copies with no evidence of duplications (**Figure 11A**). *A. trichopoda* harbored one tandem-duplicate, while *P. patens* showed four block and eight tandem duplicated *Brd*-genes (**Figure 11A**). Among the monocots, *Brd*-gene copies ranged from 14 (*A. shenzhenica*) to 79 (*T. aestivum*), and except three, all genomes showed different duplication types, a) block events (BD), b) tandem events (TD), c) both tandem and block events (TD, BD), d) tandem and combined events (TD, TD + BD), e) block and combined events (BD, TD + BD), and f) all events (**Figure 11B**). Events BD, TD and TD + BD were responsible for higher *Brd*-genes in several monocots viz. *Z*. *mays*, *M*. *acuminata*, *E*. *guineensis*, *M*. *sinesis*, *T*. *turgidum*, *S*. *spontaneum* and *T*. *aestivum* (**Figure 11B**). Likewise, dicots also showed several combinations of events (BD, BD and TD, TD + BD) leading to *Brd*-genes from 20 (*B*. *vulgaris*) to 62 (*G*. *max*). Events BD, TD and TD + BD were major contributors to higher *Brd*-genes among *D. carota, P. trichocarpa, M. esculanta, C. arietinum, B. rapa, B. oleracea, C. quinoa, P. bretscheneideri, A. chinensis* and *G. max* (**Figure 11C**). The duplication events seem to have contributed towards expansion of *Brd*-gene copies among plants.

## 4 Discussion

The chromatin state modulation mediated by epigenetic mark readers, writers and erasers is central to cellular responses towards metabolic, developmental and environmental cues (Loidl, 2004; Lauria and Rossi, 2011; Ojolo et al., 2018; Samo et al., 2021; Yung et al., 2021). Chromatin dynamics (mediated by DNA/histone modifications) is crucial for gene regulation towards adaptive responses (Strahl and Allis, 2000; Bäurle and Trindade, 2020; Bhadouriya et al., 2021), wherein epigenetic modifications of histones are important for plants response to salinity, drought, and temperature (cold/heat) stress (Kim et al., 2015; Yung et al., 2021). Studies on Brd-family of epigenetic mark readers (predominantly from animal systems) show their importance in diverse cellular functions (Tamkun et al., 1992; Zeng and Zhou, 2002; Sanchez and Zhou, 2009; Rao et al., 2014; Taniguchi, 2016; Uppal et al., 2019; Boyson et al., 2021). On the contrary, studies on plant Brd-homologs (primarily from *A. thaliana* and few other plants) are comparatively less, and include homologs like GTE4 (mitotic cell cycle and JA-mediated immune response, Airoldi et al., 2010; Zhou et al., 2022), GTE6 (leaf development, Chua et al., 2005), GTE1/IMB1, GTE8/BET9 and GTE11 (sugar and abscisic acid responses, Duque and Chua, 2003; Misra et al., 2018), GCN5 (developmental and stress response, Martel et al., 2017), and SANT-type proteins (pathogen response, Sukarta et al., 2020). However, studies on many other plants like *O. sativa* (a monocot plant system), and role of duplication (common in plant genomes) and AS-events on Brd-diversity has not been explored.

Present comparative analysis of *A. thaliana* and *O. sativa* Brd-homologs provided insights into diversity of genes/proteins/regulatory elements, orthologs and paralogs, along with duplication and AS-mediated effects on key BRD-features. Response of Brd-members to salinity is indicative of their involvement in stress-induced epigenetic regulation (Bhadouriya et al., 2021; Yung et al., 2021). Recently, GCN5 Brd-type has been reported to be involved in salt tolerance response in *A. thaliana* (Yung et al., 2021). The salinity-induced AS-modulation generated *AtBrdPG1b* alternative isoform (lacks C-ter Ser RR), which might differ in key features affecting its function/interaction (Reddy et al., 2013). As several At-/Os-Brd-homologs are affected by AS (**Figure 4**), it is important to decipher their functional significance. Furthermore, genomic duplications also contributed towards the *Brd*-gene family expansion among the plants, and hence understanding its significance in Bromodomain-diversity is important. Recently, three AtBRDs has been identified as subunits of SWI/SNF multi-protein chromatin remodeler, with role in binding of BRM-ATPase to the target genes (Jarończyk et al., 2021). Present study placed these AtBRDs to the OG3 (BD2 duplicates AtBRD3a-AtBRD3b and AtBRD3c), which also suggests similar roles for corresponding *O. sativa* homologs (OsBRD3a, OsBRD3b). Presence of multiple At- and OsBrd-members suggests their involvement in diverse cellular functions (as in humans), however, the number and domain diversity of plant homologs was substantially less (Filippakopoulos et al., 2012; Fujisawa and Filippakopoulos, 2017). Further, in both plants, the Brd-members lacked dual/poly BRD architecture like human BRDs (Sanchez and Zhou, 2009; Filippakopoulos et al., 2012), except ^BD2^OsBRDPG3c that was predicted to harbor an additional BRD-region with high heterogeneity and lack of key BRD-fold elements. Few lower photosynthetic organisms do harbor Brd-members with more than one BRD-region viz. MCO15G409l (*M. commoda*) and Cre05.g247000BRD (*C*. *reinhardtii*).

A notable feature of *A. thaliana* and *O. sativa* Brd-members was enhanced diversity due to genomic duplications, important for evolution of multi-gene families among plants (Flagel and Wendel, 2009; Barker et al., 2012; Qiao et al., 2019). In *O. sativa*, the OsBrd-duplicates displayed higher divergence, as well as different outcomes for the tandem duplication (TD) events. While, the TD1 event generated a Brd-copy in a 3-member PG3 group, another event affected the *OsBrdST2* (LOC_Os02g09920, domains: BRD-PHD-WHIM1-ZnF) and generated LOC_Os02g09910 encoding protein lacking BRD-domain (**Supplementary Figure 7**). Maintenance of single-copy Brd-members (in both species) from OG8-OG13 (GTE1 TF, GTE12 TF, GCN5, BRM, ATPase-family, **Figure 1C**), and their comparable expression levels (**Figure 7**) indicates involvement in essential conserved functions (Panchy et al., 2016). On the contrary, species-specific duplications enhanced the copies of *Brd*-genes primarily encoding for TFs of GTE-type (*A. thaliana*, OG1: GTE8; PG1: GTE3; PG2: GTE2 and *O. sativa*, PG3: GTE7), BRPF3 (*A. thaliana*, OG3) and BDF2 (*O. sativa*, OG5). Among plants, retention of duplicated genes involved in certain functions (transcription regulation, signalling, stress responses) is likely to be associated with gene-dosage imbalance or paralog interference (Panchy et al., 2016). Post-speciation duplication, and post-duplication loss can also lead to differences in copy-number of genes (Altenhoff et al., 2019; Qiao et al., 2019), and possibility of both these mechanisms cannot be ruled out for differences in AtBrd or OsBrd members. Such events may result into divergence of certain gene-copies affecting regulatory, structural and functional characteristics.

Changes in promoter sequence/structure including the CpG islands (initiates dispersed transcription initiation events, Deaton and Bird, 2011) may affect expression dynamics of duplicate *AtBrd* and *OsBrd*-genes, which may modulate the relative levels of Brd-homologs affecting the chromatin dynamics during response to metabolic and environmental cues (Lämke and Bäurle, 2017; Ojolo et al., 2018; Zhang et al., 2018; Chang et al., 2020). As epigenetic regulation is integral to plants responses to different stress conditions (Kim et al., 2015; Yung et al., 2021), differential response of certain *At-* and *OsBrds* indicates their roles as both positive and negative epigenetic modulators during stress-response. Intriguingly, several *At*- and *OsBrds* displayed AS-events, known to enhance transcriptome and/or proteome diversity (Ali et al., 2007; Reddy et al., 2013; Laloum et al., 2018). The *Brd*-genes with relatively conserved gene/protein organization seems to have evolved towards different splicing patterns. Further, if the related *Brds* of both species (including the duplicated *Brd*-members) showed AS-events, the impact on the transcript and/or protein isoforms was different (**Figure 4**). While AS-mediated differences in UTRs may affect the stability, translation, localization of transcripts (Mignone et al., 2002), events in exons can alter structural-functional characteristics. Higher abundance of AS-isoforms of certain *OsBrds* (^BD1^ *OsBrd4a.2*, *OsBrd4b.2*, ^BD3^ *OsBrd5a.2*) (**Supplementary Figure 8**) and salinity induced *AtBrdPG1b.2* (**Figure 7E**) may have some functional importance, which needs further investigation for better insights. It is reported that different duplicated genes in plants may diverge to undergo independent, functionally shared, or accelerated AS-modes (Iñiguez and Hernández, 2017). Our analysis shows that *A. thaliana* and *O. sativa Brd*-duplicates generates non-shared isoforms, indicative of evolution towards AS-mediated sub-functionalization. In a recent study AS-mediated impact on fate and interaction of two GCN5 isoforms was reported in *B. distachyon* (Martel et al., 2017). In our analysis, the GCN5 Brd-member in *A. thaliana* and *O. sativa* belong to OG10 (AtBRD10, OsBRD10, **Figure 1C-x**), show similar domain organization (**Figure 3**) but lack AS-events (**Figure 4**), indicating absence of AS-mediated functional diversification like *B. distachyon.* Detailed analysis of AS-events in Brd-homologs in both the plants is worth investigating.

Structural variations in the BRD-fold are known to alter its interaction with acetylated lysine on histones, and associated functions of the Brd-proteins (Josling et al., 2012). The At- and OsBRD-members (including duplicates) harbored variations (substitutions at key sites, additional secondary elements, and partial/complete loss of BRD-fold elements), which might affect their interaction capability/affinity with the chromatin. It is therefore important to decipher their structural-functional characteristics vis-à-vis other BRD-members. BRD-region similar to OsBRD3a and OsBRDST2 with characteristic long N-ter deletion (caused loss of αZ-helix, ZA-loop) was not observed among AtBRDs, however an uncharacterized human protein showed similar deletion and loss of elements (**Supplementary Figure 6**). Interestingly, the At- and OsBrd BRD-fold elements harboured several conserved signatures (e.g., leucine repeat pattern in αZ and sites in ZA-loop) suggesting similar roles in interaction with other helices/loops, as reported in human-BRDs (Filippakopoulos et al., 2012). Plant-specific amino acid variations in αB, αC and ZA loop (particularly among BRD-duplicates) are also likely to affect their interaction with chromatin, and associated functions. The consistency between the BRD-region based relationships, and ortholog-paralog clustering, show its utility in deciphering the divergence of Brd-family in a species, and to overcome issues related to the analysis of such multi-domain proteins (Nakano et al., 2006). Although, the At/OsBRD-homologs lacked dual-BRDs like certain human BRD-homologs (Filippakopoulos et al., 2012), similar domains were identified among different At/OsBRDs, and it would be interesting to find out if they differ in their interaction capabilities (Miller et al., 2016).

Contribution of genomic duplications, known to enhance the copy number and/or diversity of plant genes (Qiao et al., 2019), was also evident in *Brd*-gene copy number in most plants analyzed. Duplication of *Brd*-genes was not evident among lower photosynthetic organisms (*M. commoda*, *S. moellendorffii*, *C. reinhardtii*, *M. polymorpha*). The *Brd*-gene copies increased in *A. trichopoda* (single genome duplication event, Amborella Genome Project, 2013) and *P. patens* (two whole genome duplication events, Lang et al., 2018). Interestingly, without duplications *P. abies* contain higher *Brd*-genes, which might be associated with inherent transposon activity, and large genome size (Nystedt et al., 2013). Among higher plants, more duplication events have contributed towards higher gene copies (Qiao et al., 2019). Monocots affected by multiple duplication events (ζ, ancestral; ϵ, paleohexaploidization; σ and ρ, predating Poaceae divergence), lineage-specific events (*M. acuminata*), and polyploidy (*T. turgidum*, tetraploid; *T. aestivum*, hexaploid) (Wang et al., 2017) harbor higher *Brd*-gene copies. Likewise, the dicots affected by primitive duplications (ζ, ϵ), triplication (WGT, γ), and lineage-specific WGD/ploidy events (α and β, crucifer lineage; *Gossypium*-specific ploidy; WGDs specific to poplar, legumes, *Glycine*) (Wang et al., 2017) also showed higher *Brd*-gene copies. Moreover, *Brd*-gene copies might also be affected by post-duplication losses/deletions (Qiao et al., 2019), and is likely in plants like *A. shenzhenica*, *P. equestris*, *Z. marina*, which lack *Brd*-duplicates despite an ancient WGD event (Cai et al., 2015; Olsen et al., 2016; Zhang et al., 2017).

The present analysis revealed extensive diversity among important aspects of *A. thaliana* and *O. sativa* Brd-members. Functional aspects are likely to be conserved among Brd-orthologs maintained as single copy in both species viz. TF GTE1 (OG9), GCN5 (OG10), BRM, ATP-dependent helicase (OG11), GTE12 (OG12), ATPase family-AAA domain (OG13), and an uncharacterized BRD (OG8). In both the species, genomic duplications and alternative splicing have contributed towards the Brd-homolog diversity. Species-specific evolutionary trends were also identified in the two species, like generation of four extensively diverse *AtBrds* due to two block duplications in OG2 (compared to single OsBrd-member), and unequal number of *Brds* due to duplication events in species-specific PGs (PG1, PG2 and PG3), most of which are still not completely characterized. The *Brd*-gene copies were substantially enhanced among several complex photosynthetic organisms with history of duplication events. Overall, the plant *Brd*-gene family is relatively less studied, however its diversity, impact of duplication and AS-events, domain signatures, suggest involvement in diverse cellular mechanisms, which advocates a thorough analysis for understanding their functional significance.

## Data availability statement

The datasets presented in this study can be found in online repositories. The names of the repository/repositories and accession number(s) can be found in the article/**Supplementary Material**.

## Author contributions

TVA: analysis of gene/proteins, RNA-Seq data, and sequence divergence; RPS: analysis of transcripts and domain heterogeneity; HSM: data analysis and review, manuscript writing; AS: planning and execution, in silico and experimental analysis, data review, manuscript compilation and communication. All authors contributed to the article and approved the submitted version.
