# Supplementary material for: Genome-wide analysis of bromodomain gene family in Arabidopsis and rice
Source: Front Plant Sci. 2023 Mar 8;14:1120012. doi: 10.3389/fpls.2023.1120012 (PMC10030601; doi:10.3389/fpls.2023.1120012)

Supplementary Figure 1: Schematic representation of alternative splicing events in *Brd*-genes of *A. thaliana* (A) and *O. sativa* (B), belonging to different ortholog groups (OGs), paralog groups (PGs), and singleton category (STs). Block duplicated genes (BD), constitutive transcript (.1), alternative transcripts (.2 to .6), UTRs (white boxes), exons (dark grey boxes) and introns (dashed lines) are indicated in the figure. Scale on the top indicates the length of transcripts (kilobase, kb).

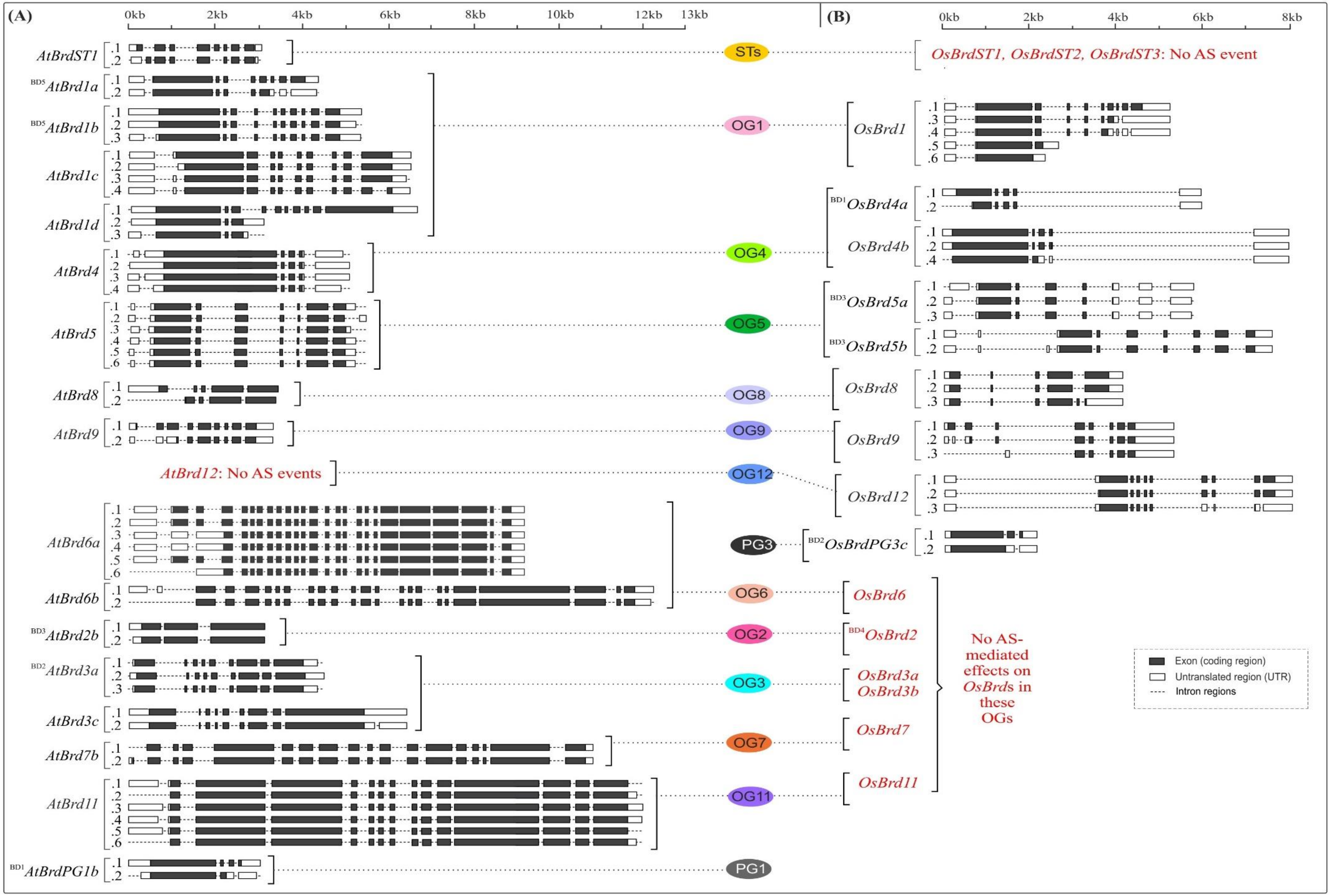

Supplementary Figure 2: Motif heterogeneity among BRD-proteins of *A. thaliana* (A) and *O. sativa* (B), belonging to thirteen ortholog groups (OG1-13), three paralog groups (PG1-3), and singleton category (STs). Motifs M1-M15 are shown in different color codes. Scale on the top indicates the protein length (number of amino acids). Duplicate BRD-pairs are indicated with the designations 'BD' (block duplication) and 'TD' (tandem duplication) in the names.

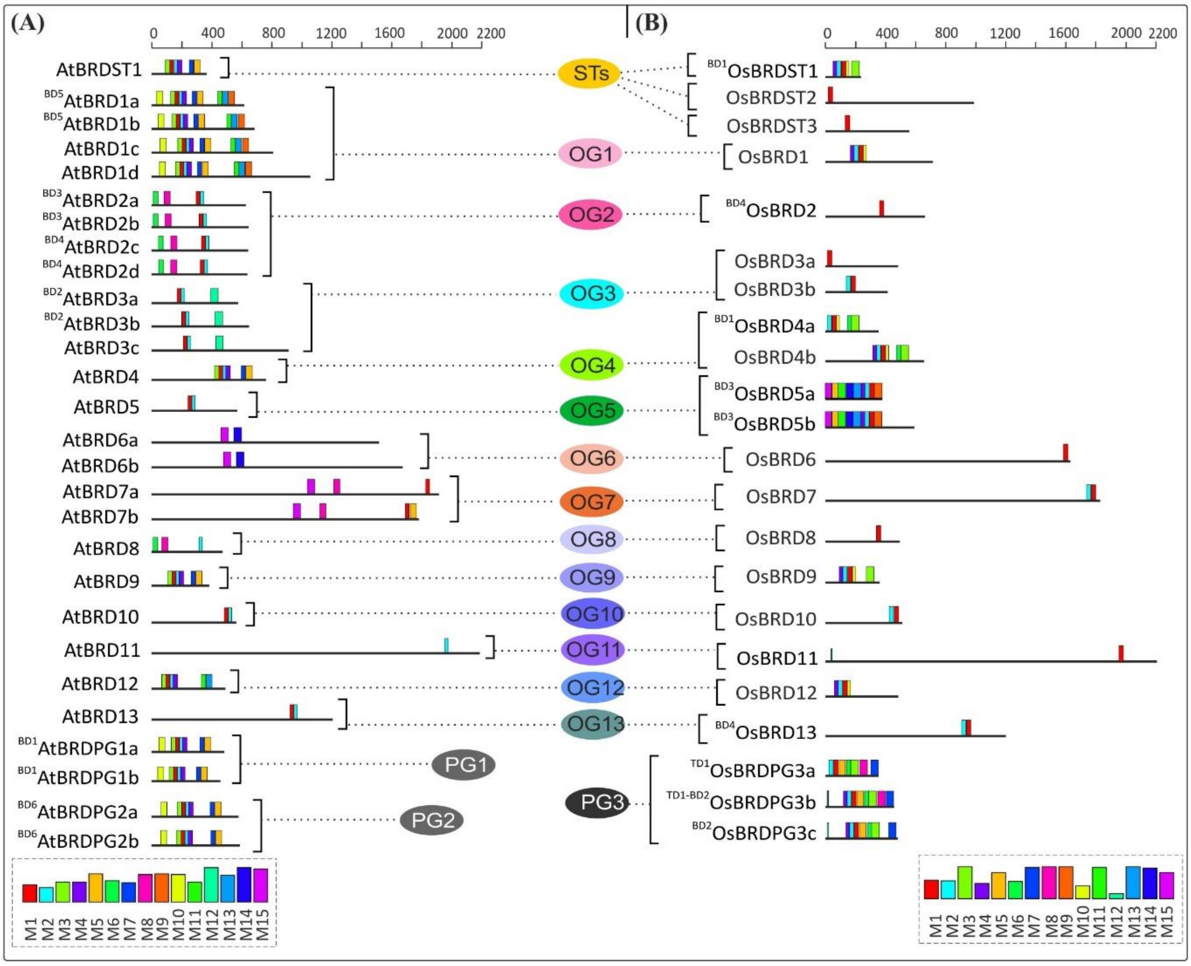

Supplementary Figure 3: Diversity of *cis*-elements (as per analysis at PlantCARE database) in the upstream regions of *A. thaliana Brd*-genes belonging to thirteen ortholog groups (OG1-13), two paralog groups (PG1-2), and singleton category (STs). Different types of elements are indicated by different symbols/colours, *cis*-elements belonging to six major functional categories are indicated below, and block-duplicated genes are indicated by the designation 'BD' in the gene name. Scale on the top indicates the length in kilobase.

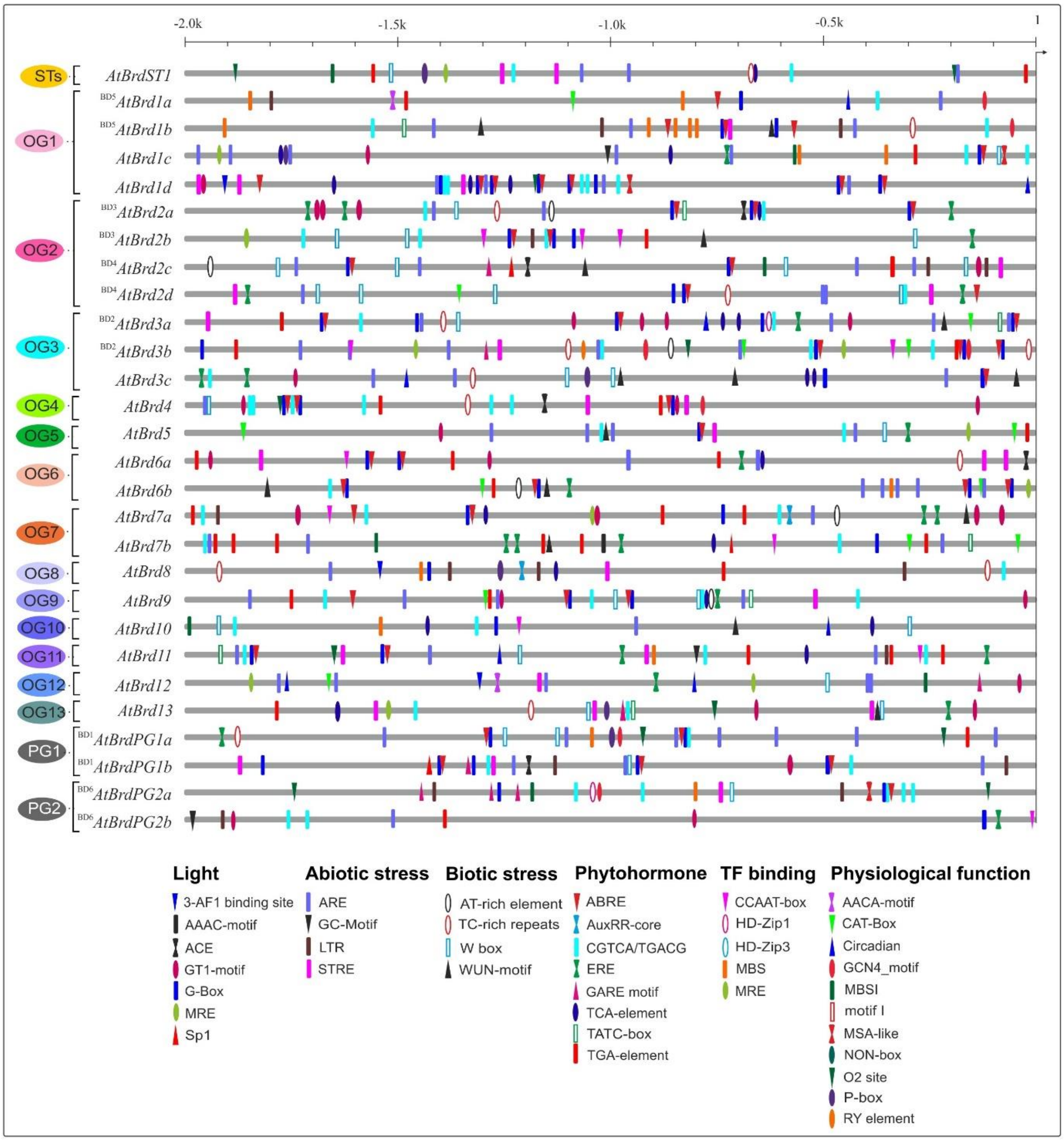

Supplementary Figure 4: Diversity of *cis*-elements in the upstream regions of *O. sativa Brd*-genes belonging to thirteen ortholog groups (OG1-13), one paralog group (PG3), and singleton category (STs), as per analysis at PlantCARE database. Different types of elements are indicated by different symbols/colours, *cis*-elements specific to six functional categories are listed below, and genes affected by block or tandem duplications are indicated by the designation 'BD' or 'TD' in the gene names. Scale on the top indicates the length in kilobase.

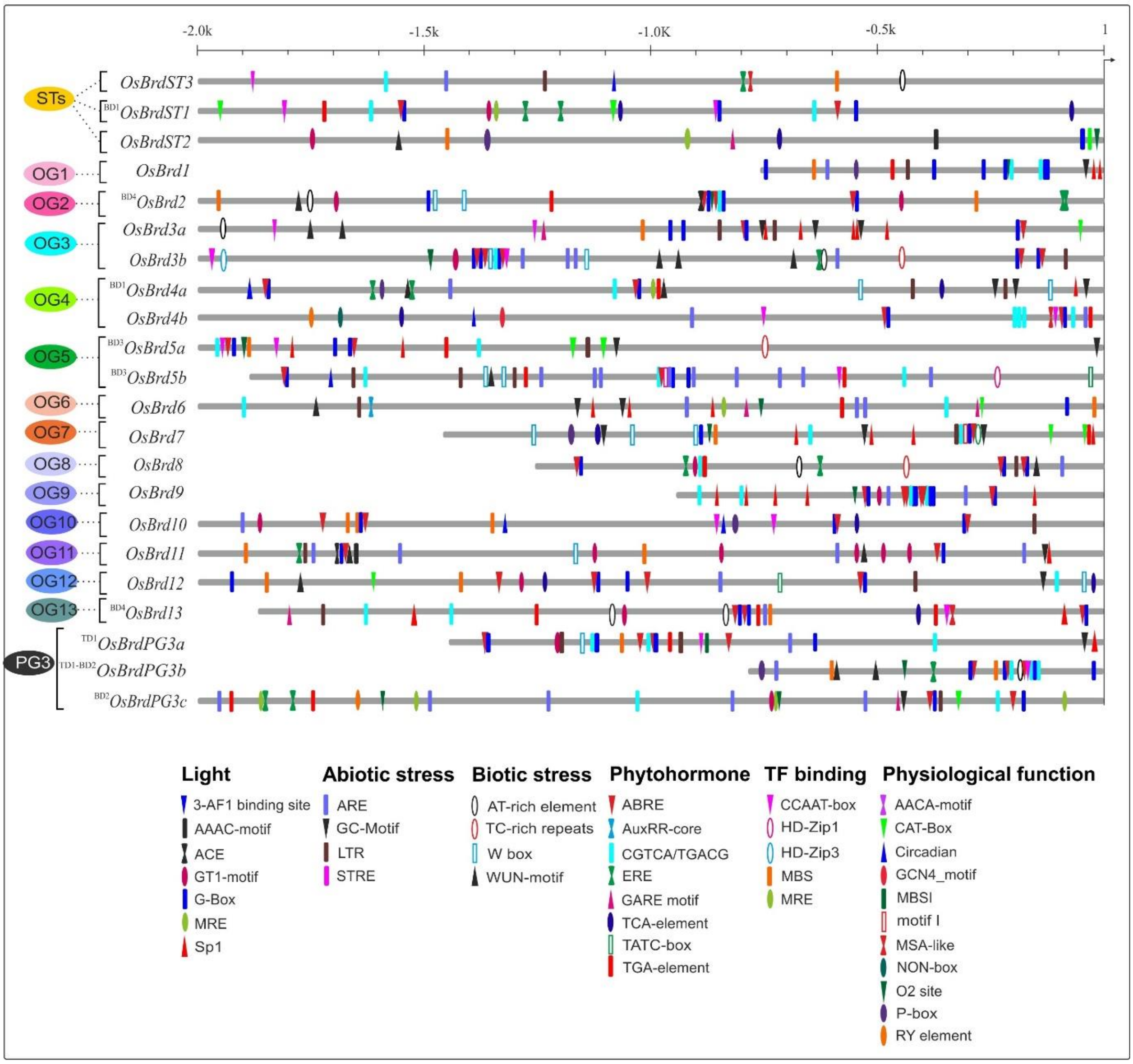

Supplementary Figure 5: Multiple sequence alignment of the bromodomain-region of 28 AtBRD and 22 OsBRD-homologs. Scale on the top indicates the sites in the MSA file. The duplicate-pairs are indicated with designations ‘BD’ (block duplication) and ‘TD’ (tandem duplication) in names, while numerals (I-VI) indicates the six major clusters as per phylogenetic analysis (Figure 9A).

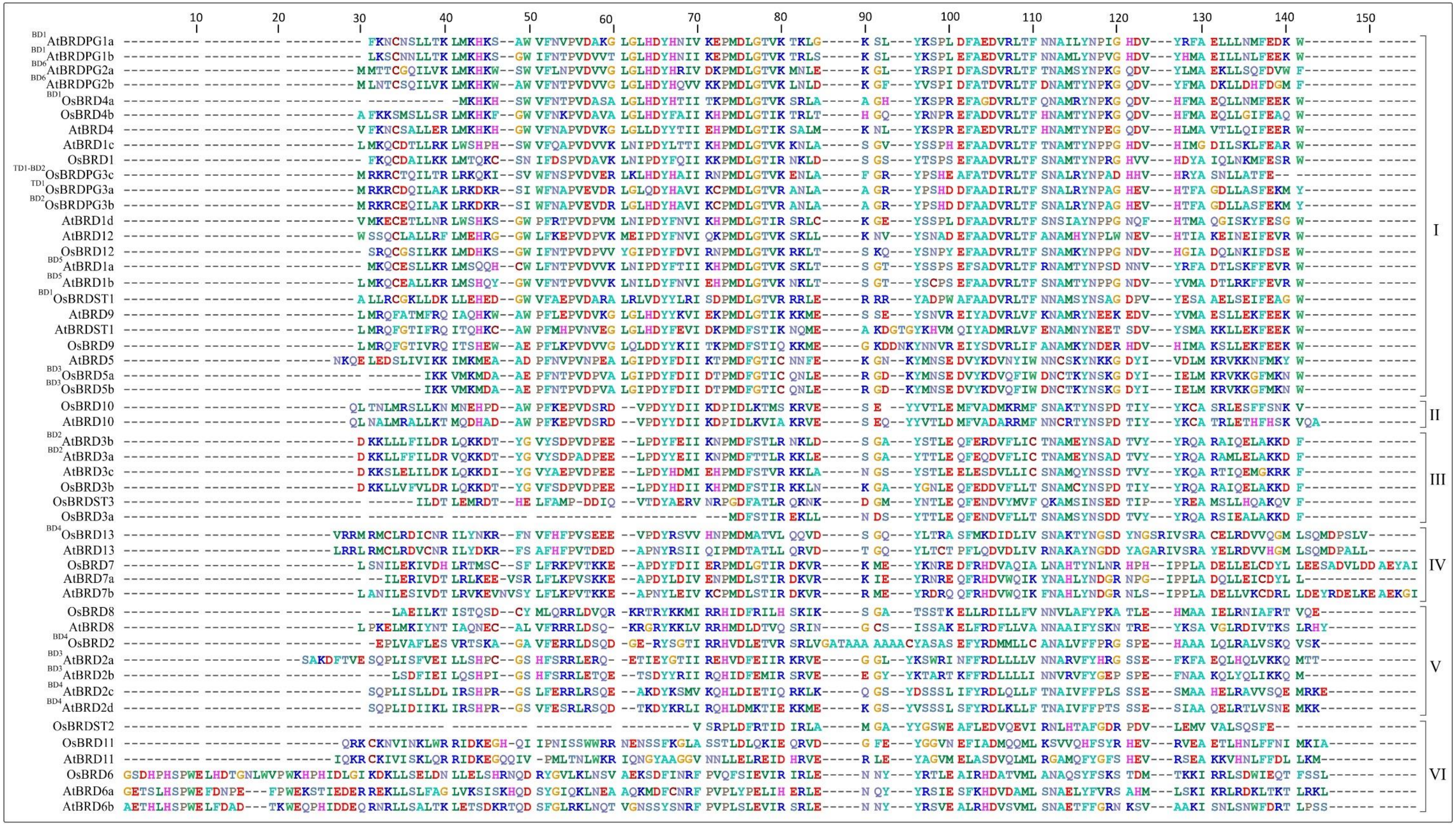

Supplementary Figure 6: (A) Homology model of a normal bromodomain (BRD) region containing all typical structural features of the BRD-fold (four α helices: αZ, αA, αB, αB and three loops: ZA, AB, BC). (B) Homology model of BRD-region of a human protein (K2026_Human, UniProt ID: Q5HYC2) with a long deletion at N-terminal region (similar to deletion in OsBRD3a and OsBRDST2), leading to loss of αZ and ZA-loop elements.

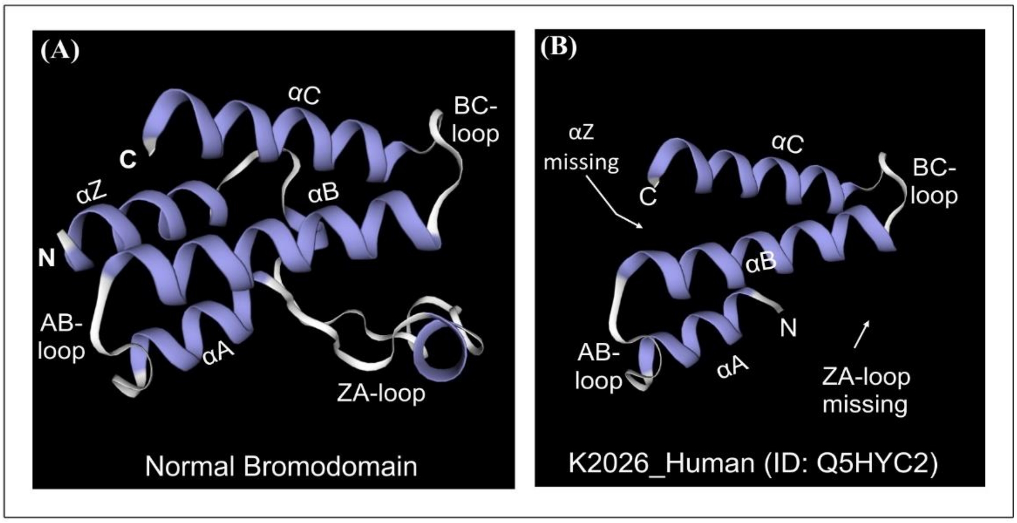

Supplementary Figure 7: CDD-NCBI based conserved domain analysis of OsBRDST2 (LOC_Os02g09920, BRD-homolog with BRD-PHD-WHIM1-ZnF domain combination (A) and its tandem duplicate gene locus (LOC_Os02g09910) encoding protein contaning only PHD domain (B).

(A)

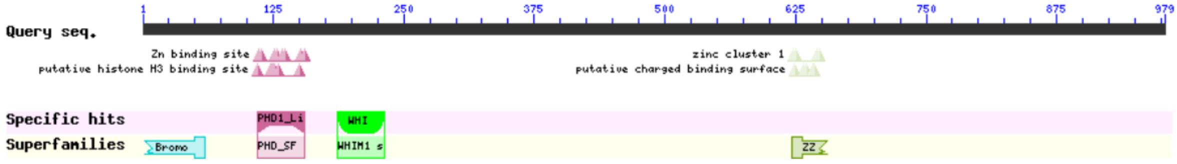

(B)

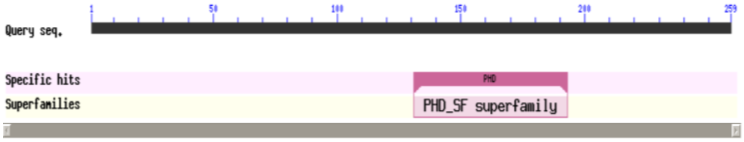

Supplementary Figure 8: Heatmap-based analysis of RNA-Seq data of constitutive and alternative transcripts of *OsBrd*-genes in different tissues (A) and stress conditions (B). The *OsBrd*-transcript designations (constitutive transcript: .1; alternative transcripts: .2 to .5) are mentioned on the left side of the heat maps, tissues and stress conditions are listed on the top, and a gradient color scale indicating expression level from blue (low) to red (high) is shown on the bottom of the figure.

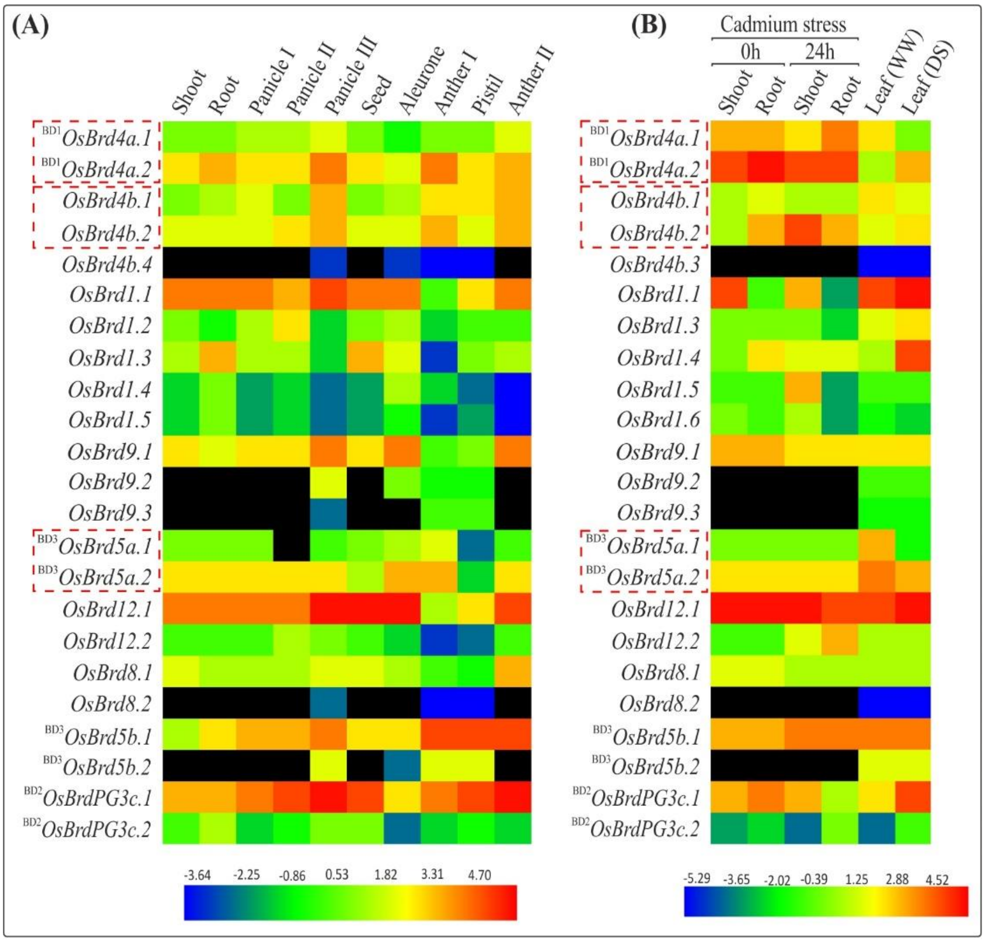

Supplement: Supplementary file 1 [file DataSheet_1.pdf]
