# Supplementary material for: Genome-wide analysis of bromodomain gene family in Arabidopsis and rice
Source: Front Plant Sci. 2023 Mar 8;14:1120012. doi: 10.3389/fpls.2023.1120012 (PMC10030601; doi:10.3389/fpls.2023.1120012)
Supplement: Supplementary file 2 [file DataSheet_2.pdf]

Supplementary Table 1: Oligonucleotide primers used for RT-qPCR analysis of *AtBrd* and *OsBrd* genes.

S. No.	Primer Name	Gene	Primer Sequence (5´-3´)
1	AtBrd1a-ForP	^{BD5}*AtBrd1a*	GGCATTGCTTGAGATGGAGAAGTCGGTTG
2	AtBrd1a-RevP	(AT3G01770)	CAAATACAGCCAACCCATCAGACTCAGAGCC
3	AtBrd1b-ForP	^{BD5}*AtBrd1b*	GCCAGGCATTGATGGAGATGGAGCAGTCAG
4	AtBrd1b-RevP	(AT5G14270)	ACCCAACATCAGGCCCATCCTCCTCCTC
5	AtBrd3a-ForP	^{BD2}*AtBrd3a*	TAAAACAATTAACTCCAGTTGGACTGAAAGCTGAATATGGGT
6	AtBrd3a-RevP	(AT1G76380)	CAACCCACCCGGGACCAAATTCGGTTCCA
7	AtBrd3b-ForP	^{BD2}*AtBrd3b*	GAGTTAAAACAGTTGATTCCAGTTGGGCTGAACATGGAG
8	AtBrd3b-RevP	(AT1G20670)	GGGCAATACAGTCTCGATTCTCCTCGATGC
9	AtBrdPG1a-ForP	^{BD1}*AtBrdPG1a*	AGTTACTGAATCAGGAAAAGC
10	AtBrdPG1a-RevP	(AT1G17790)	GTCACTGTCAGTATCACTAGAG
11	AtBrdPG1b-ForP		GTCATGTGGCATCTACTGTTC
12	AtBrdPG1b-RevP		AGCTATCACTGTCAGAGCCACTA
13	AtBrdPG1b.1-ForP	^{BD1}*AtBrdPG1b*	AAGTTACTGAATTAGGTCATG
14	AtBrdPG1b.1-RevP	(AT1G73150)	AGCTATCACTGTCAGAGCCACTA
15	AtBrdPG1b.2-ForP		TGTTTTTGACACTTGAAAAG
16	AtBrdPG1b.2-RevP		AGCTATCACTGTCAGAGCCACTA
17	AtBrd2a-ForP	^{BD3}*AtBrd2a*	GACTCGAACGTCAGGAAACAATTG
18	AtBrd2a-RevP	(AT2G42150)	AACTCTGAAGATCCTCTGTG
19	AtBrd2b-ForP	^{BD3}*AtBrd2b*	GTAACCTGGTCTCGCCAGGATC
20	AtBrd2b-RevP	(AT3G57980)	TTCAACTGCAATGACGAGATGG
21	AtBrd2c-ForP	^{BD4}*AtBrd2c*	AGGGTGGTGCCGGCGAGATC
22	AtBrd2c-RevP	(AT2G44430)	AATCCTTAGTGTCCTGGCTC
23	AtBrd2d-ForP	^{BD4}*AtBrd2d*	AGAGGAAAGGACAGAAATAC
24	AtBrd2d-RevP	(AT3G60110)	AATCCTTAGTGTCCTGGCTC
25	AtBrdPG2a-ForP	^{BD6}*AtBrdPG2a*	CTCCACCAAGAAACATGCCTCCGG
26	AtBrdPG2a-RevP	(AT5G10550)	TAGAGCCACCACTAGCAGCGGCAGCTG
27	AtBrdPG2b-ForP	^{BD6}*AtBrdPG2b*	CTCCACCTAGGAACATGGCTTC
28	AtBrdPG2b-RevP	(AT5G65630)	CACTACTAGCAGCAGCTGCAA
29	AtAct-ForP	*AtActin*	CACAGCACTTGCACCAAGCAGCAT
30	AtAct-RevP	(AT3G18780)	TGGAGATCCACATCTGCTGGAATG
31	OsBrd4a-ForP	^{BD1}*OsBrd4a*	ATGATAATGAGAGGTATGTAGGCTCATCATCACC
32	OsBrd4a-RevP	(LOC_Os01g11580)	CACTAGACGAAGAGCCTGAATCGC
33	OsBrdST1-ForP	^{BD1}*OsBrdST1*	CGGGATGATGGAGGTGGACCTGGA
34	OsBrdST1-RevP	(LOC_Os01g46040)	TCATGGCTCCTGCTTCACCTTAACC
35	OsBrdPG3a-ForP	^{TD1}*OsBrdPG3a*	CCGCTACGATGGTGGACAACGGTGATGTGACG
36	OsBrdPG3a-RevP	(LOC_Os04g53130)	GGGCGGACCCAGGGCCAGGACA
37	OsBrdPG3b-ForP	$^{TD1\text{-}BD2}$*OsBrdPG3b*	ACAATGGCAATAGAGAGCAAGGATCCTGA
38	OsBrdPG3b-RevP	(LOC_Os04g53170)	CCTCAGTATCCTTCTCAATCTCCACTGATTGGT
39	OsBrdPG3c-ForP	^{BD2}*OsBrdPG3c*	GATATTGGCGATGAGATGCCGACGGCA
40	OsBrdPG3c-RevP	(LOC_Os08g09340)	GAGCTCCTCGAGTCAGAATCACTGGATG
41	OsBrd5a-ForP	^{BD3}*OsBrd5a*	AGAGCTTATGAAACGGGTGAAGAAGGGCTTCA
42	OsBrd5a-RevP	(LOC_Os06g24870)	TCATCGCCTGTGTTATCATTGCCACCATTCTCCTG
43	OsBrd5b-ForP	^{BD3}*OsBrd5b*	GAGGATGAAGGCTCCAGACCATACTTTGACG
44	OsBrd5b-RevP	(LOC_Os08g01794)	TGGCTTGTTTCTGTTGGCTCCTTATGTTGG
45	OsBrd2-ForP	^{BD4}*OsBrd2*	GAAATACGATCTCTCGATCGGGTCGTTGCAATCA

46	OsBrd2-RevP	(LOC_Os08g39980)	CGCCGGCCTCCTCCGGTGATCC
47	OsBrd13-ForP	BD4*OsBrd13* (LOC_Os09g33980)	CGCCTGTTGCTCAGCTGGTATCTG
48	OsBrd13-RevP		TGGTGGATTGTCAGTTTCTTTCAGAACACC
49	OselF1α-ForP	*OselF1α* (LOC_Os03g08010)	GCTGCAACAAGATGGATGCCACCA
50	OselF1α-RevP		GAAGGGAATCTTGTCAGGGTTGTAGC

Note: The locus numbers of the *AtBrd*-genes are as per The Arabidopsis Information Resource (TAIR, https://www.arabidopsis.org/) and of *OsBrd*-genes, as per Rice Genome Annotation Project (RGAP, http://rice.uga.edu/).

Supplementary Table 2: List of conserved motifs identified among twenty-eight *Arabidopsis thaliana* bromodomain (BRD) containing proteins at per analysis using MEME suite software.

Motif	Motif Sequence	E-Value	Sites	Width	Functional Annotation
M1	EJPDYYNIIKHPMDLGTIKKKLEKG	3.10E-213	24	25	BRD
M2	YSSPLEFAADVRLTFNNAMTY	1.10E-166	24	21	BRD
M3	MKQCETLLRKLMKHKHGWVFNTPVDVVGL	1.90E-134	12	29	BRD
M4	NPEGNDVYVMAEKLLKLFEERWKTIEKKY	3.80E-87	12	29	BRD
M5	QIVKKSNPELSQGDDEIEJDIDALDDETLWELRRF VDEYLK	9.30E-83	12	41	ET
M6	QTWGTWEELLLACAVKRHGTGDWDSVASEVQ	2.80E-47	5	31	Myb-like DBD
M7	EPAKRDMTDEEKRKLGEDLQSLPPDKLZ	2.10E-33	11	28	ET
M8	PWLEELRKLRVAELRREVERYDLSINSLQLKVKK LEEERE	3.90E-32	7	40	-
M9	AAEAKRKRELEREAARQALLEMEKSVEINENSRF LEDLELL	2.20E-29	4	41	-
M10	DGVSKMVLSLSGLSSSERRELKRRLKSELEZVRSL RKRIE	9.40E-28	8	40	BRD
M11	PEKRYRAAJLKNRFADIILKAREKPLNQN	5.70E-25	5	29	-
M12	PVGLNAEYGYARSLARYAANLGPVAWKIASQRI EKVLPSGIKFGRGWVGE	4.30E-27	3	50	-
M13	KGDPEKLQREREELELQKKKEKARLQAEAKAAE EARRKA	1.60E-24	5	39	-
M14	ICVWNAADGSLVHCLTGHSESSYVLDVHPFNPRI AMSAGYDGKTIIWDIW	2.10E-21	2	50	WD
M15	WFLHDNFVTCSRDGRANIWRPEIRGVGGPSGRGL GFYHLKVPPPPLPP	4.10E-29	4	48	TFIID, subunit TAF1

Analysis of conserved motifs was carried out online using MEME software, available at MEME online Suite (version 5.4.1, http://meme-suite.org/tools/meme, Bailey et al., 2015).

Supplementary Table 3: List of conserved motifs identified among twenty-two *Oryza sativa* bromodomain (BRD) containing proteins, as per analysis using MEME suite software.

Motif	Motif Sequence	E-Value	Sites	Width	Functional Annotation
M1	YGSLEEFAADVRLTFSNAMTYNPKGHDVH	9.10E-187	22	29	BRD
M2	DVVALGJPDYFDIIKKPMDLGTIRKKLE	2.00E-145	15	28	BRD
M3	LPPEKLDNVLQIVKKRNGSPELVGDEIELDIDEMD VETLWELDRFVANYK	6.80E-84	7	50	ET
M4	LKRCGEILKKLMKHKAAEPFNTPV	2.30E-27	9	24	BRD
M5	ATFENMYKPAHSWFEQEPKILEPPMPVPPPPEPEK PAPSTV	4.40E-23	5	41	-
M6	MKKPKAREPNKREMTLEEKNKLRVGLZ	1.10E-20	5	27	-
M7	YVDIGDEMPTATYQSVEIEKDTEAASSGSSSSSDS GSSKDSVSESGNAH	3.90E-15	3	49	-
M8	IVNGENADVIDASVANDSDMLVNGSTATMVDNG DVTMAIESKDPDKITTQ	5.10E-13	2	50	-
M9	MKRVKKGFMKNWLAAGLYSDVQENGGNDNTG DEDVKGSKGKSKQKRRRLG	4.00E-11	2	50	-
M10	MAEQLLEIFEAKWPEIEAKV	6.10E-07	6	20	BRD
M11	VQNAKPKVYSRVRLKFKSAKVLETHQGPSEAKA PVDGGGGKPASAAPEA	5.70E-06	2	49	-
M12	HHHHHHQW	9.00E-06	3	8	-
M13	NQDRKADSVSEPLPSKQETVLENVESETALEPRSS QELEVKQATPERQRD	5.00E-04	2	50	-
M14	VAEKAIVSPDGQKDAQAAELSGSDKDKMARKVA SIKIKSVGLSSVEDK	4.90E-02	2	48	-
M15	MKRKRGRKMGKKGKLKASITADASPMSPSSPST VDASSKSP	9.80E-01	2	41	-

Analysis of conserved motifs was carried out online using MEME software, available at MEME online Suite (version 5.4.1, http://meme-suite.org/tools/meme, Bailey et al., 2015).
